# Supplementary material for: Single-cell transcriptomic analysis identifies murine heart molecular features at embryonic and neonatal stages
Source: Nat Commun. 2022 Dec 27;13:7960. doi: 10.1038/s41467-022-35691-7 (PMC9794824; doi:10.1038/s41467-022-35691-7)
Supplement: Supplementary file 1 — Supplementary Information [file 41467_2022_35691_MOESM1_ESM.pdf]

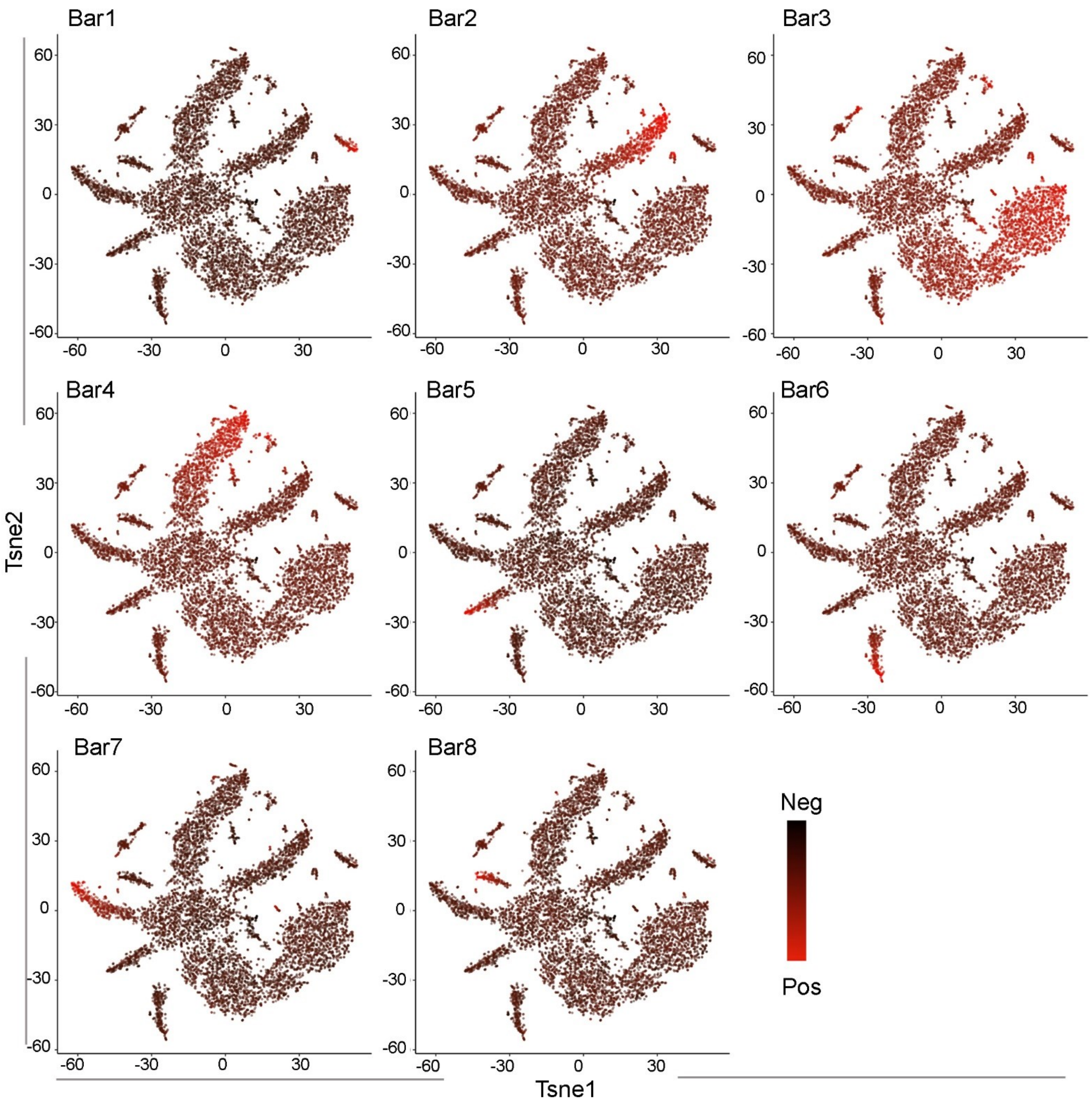

Fig S1. The expression pattern of MULTI-seq barcodes in the E18_P1 dataset. The red signal represents high enrichment, and the black signal represents low or negative signal.

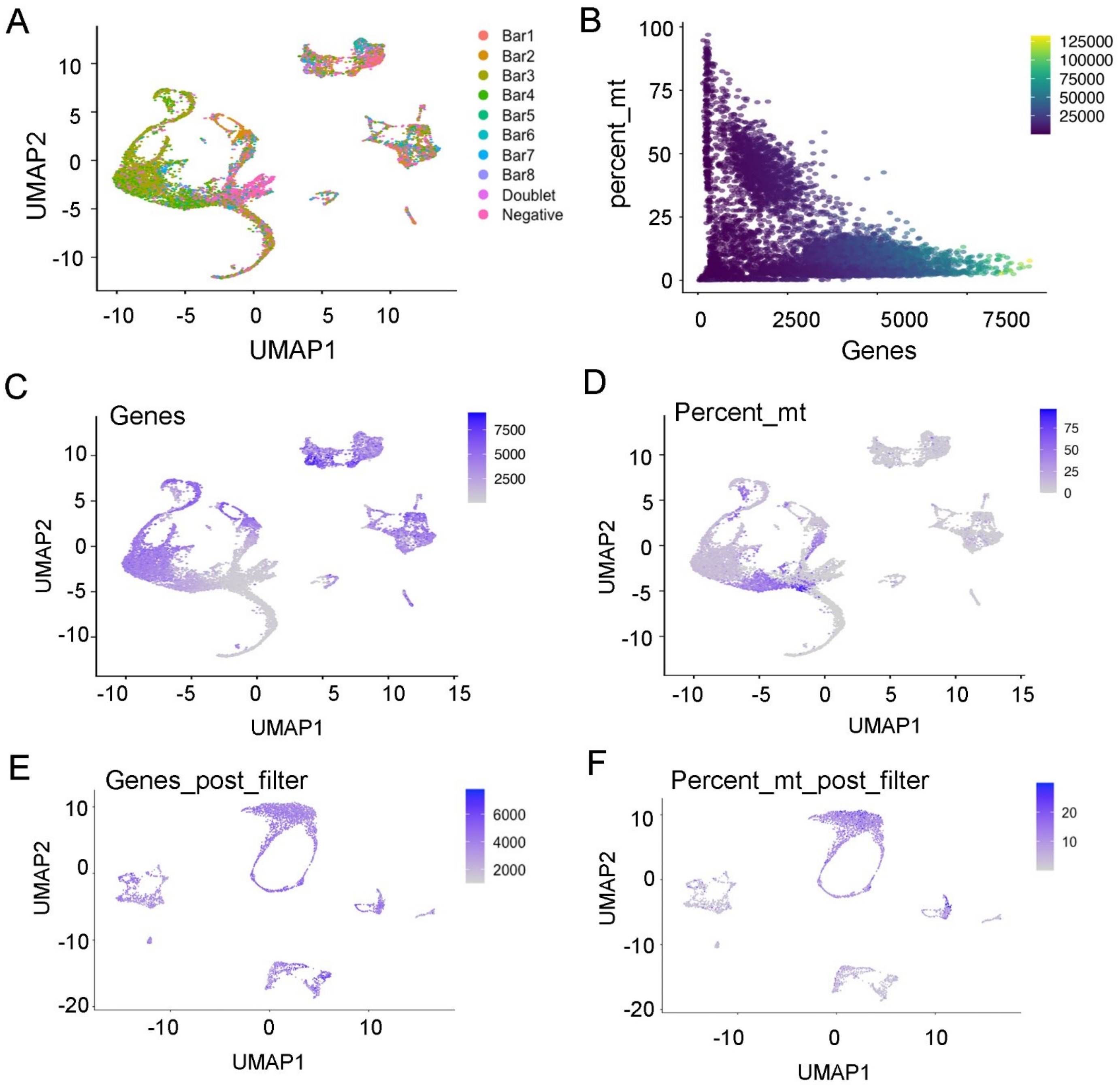

Fig S2. Quality control analysis of the E18_P1 dataset. (A) Identification of singlets, doublets, and negative cells in the E18_P1 dataset. (B) Analysis of the number of expressed genes (x-axis), the percentage of mitochondria genes (y-axis), and the total number of molecules detected in each cell (color). (C, D) UMAP plots of the gene numbers and mitochondria gene percentages in each single cell before filtering. (E, F) UMAP plots of the gene numbers and mitochondria gene percentages in each single cell after filtering.

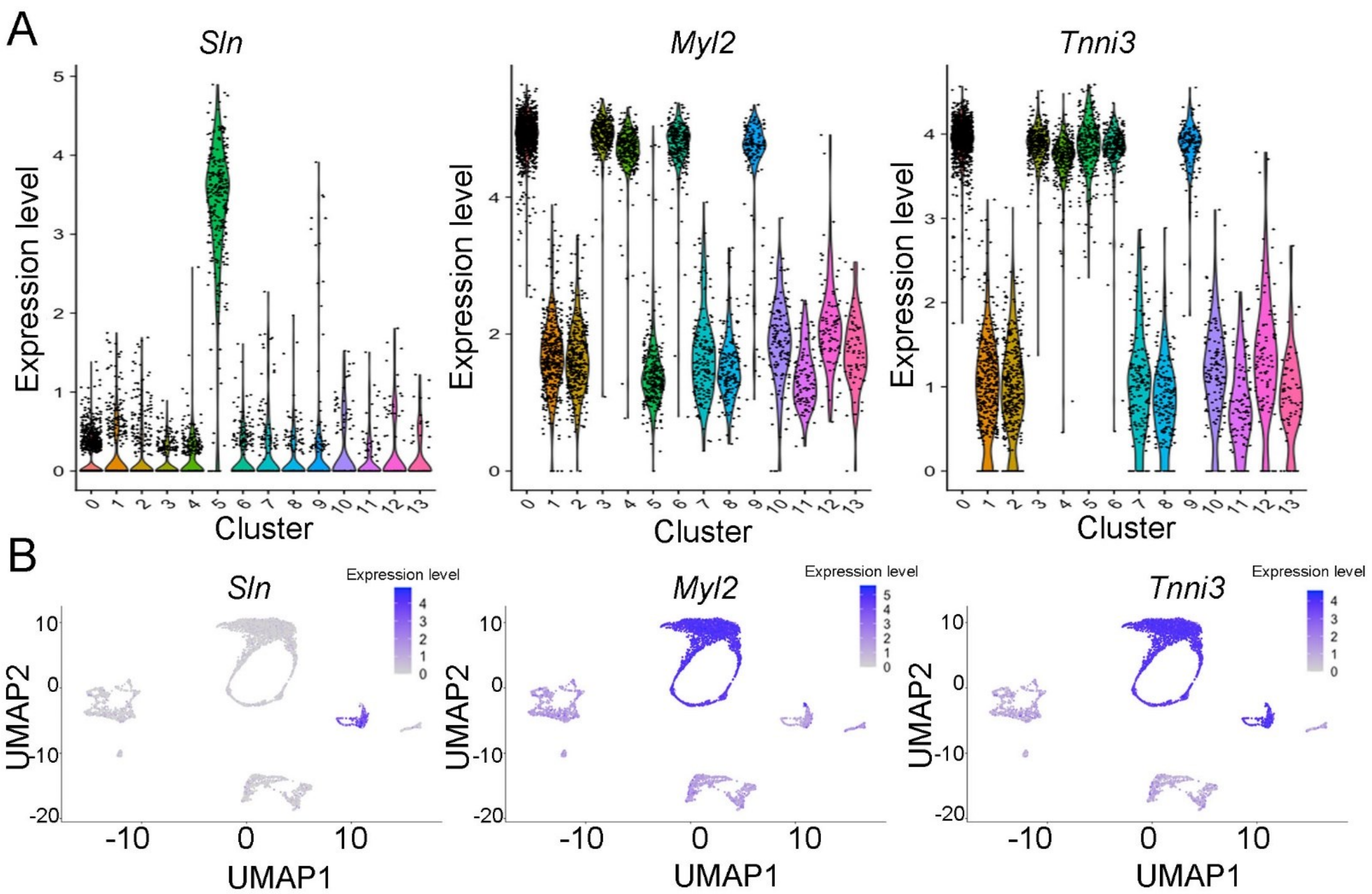

Fig S3. The expression pattern of *Sln* and *Myl2* in E18_P1 single cells. (A, B) Violin plots and Feature plots show that the clusters with atrial and ventricular CMs (*Tnni3* positive) highly express *Sln* and *Myl2*, respectively.

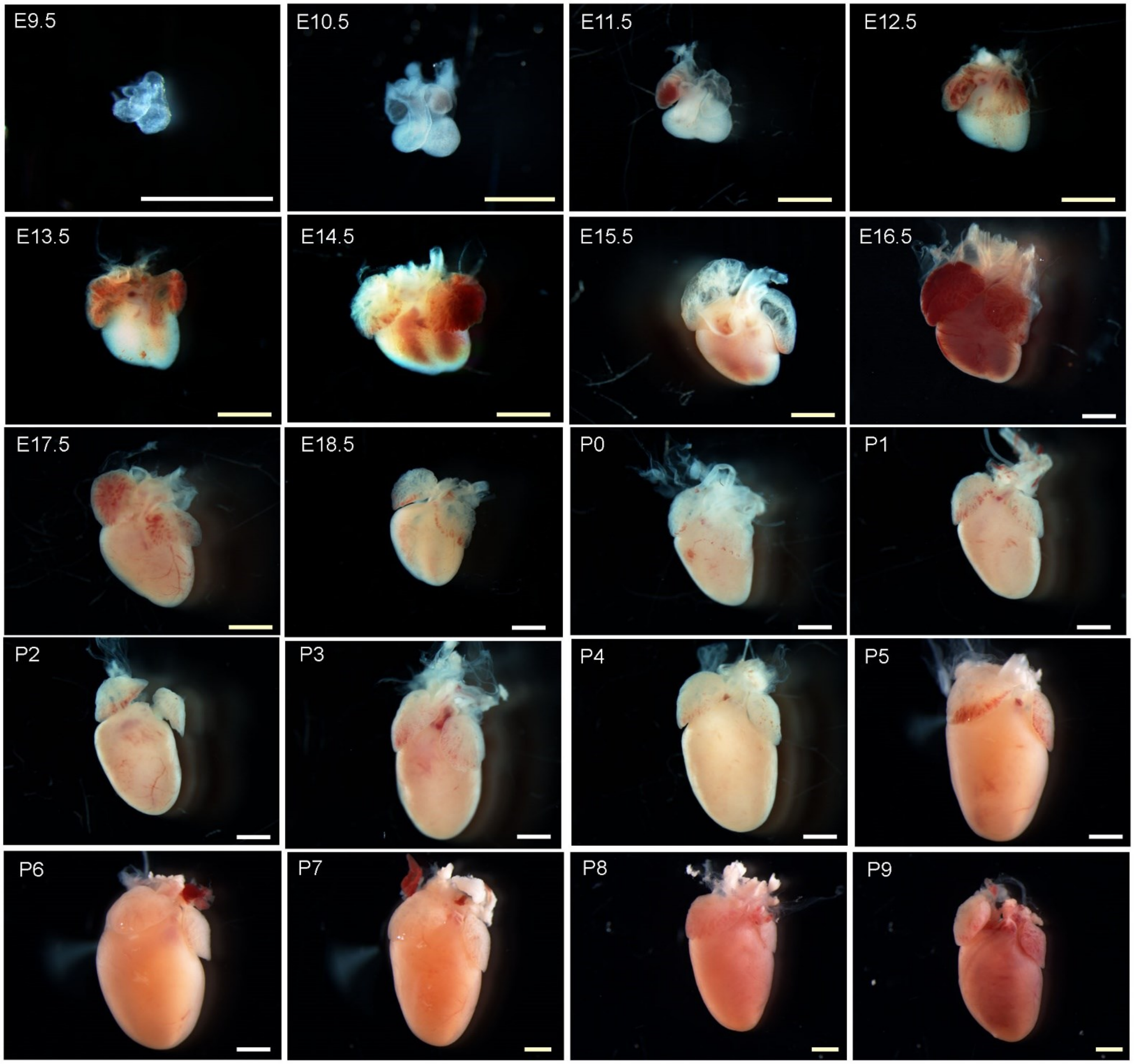

Fig S4. Representative images of staged hearts. Scale bar=1mm.

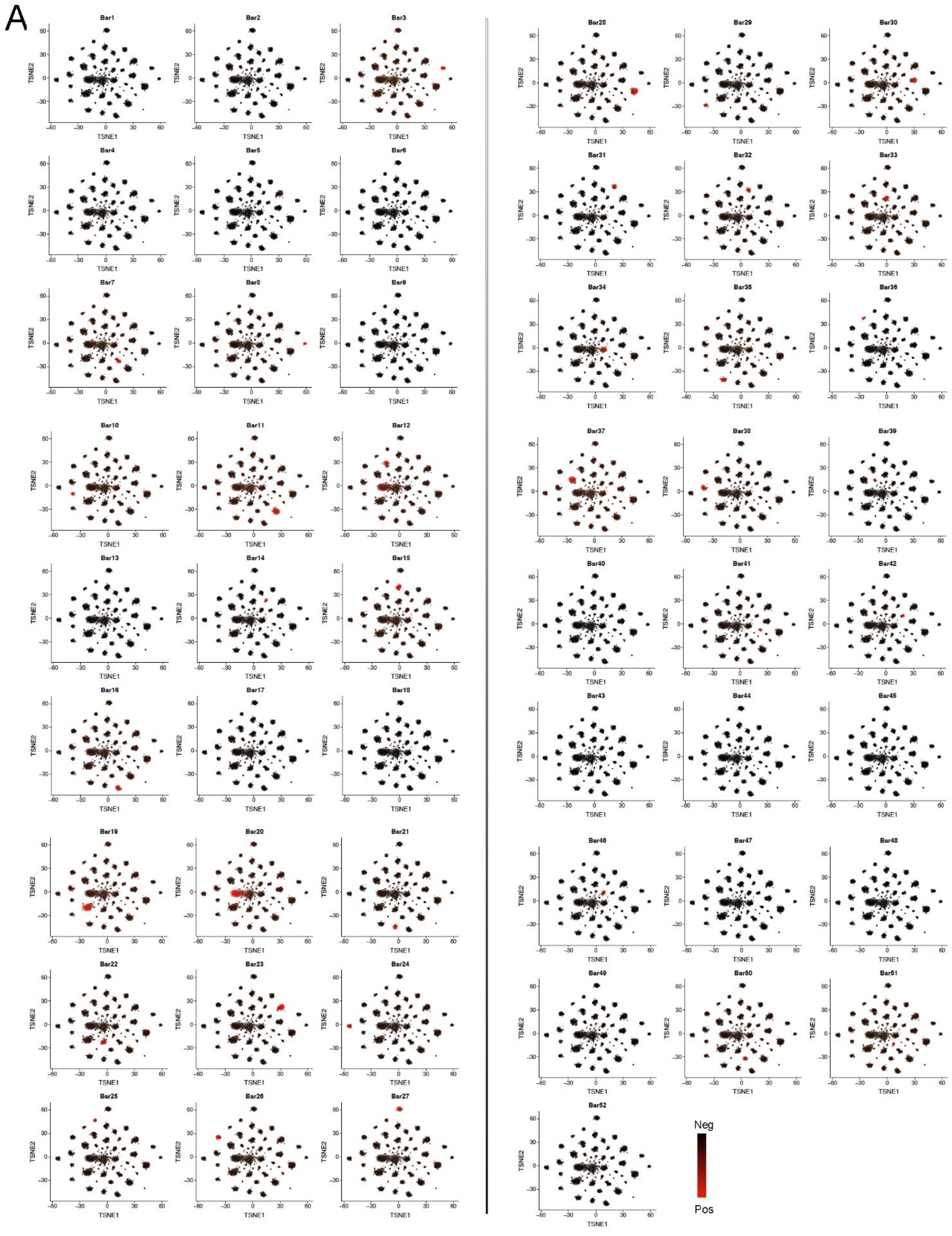

Fig S5. The expression pattern of MULTI-seq barcodes in the 5k_1 dataset. Red color represents a positive signal, and black represents the background. Each barcode was mainly enriched in one cluster of cells.

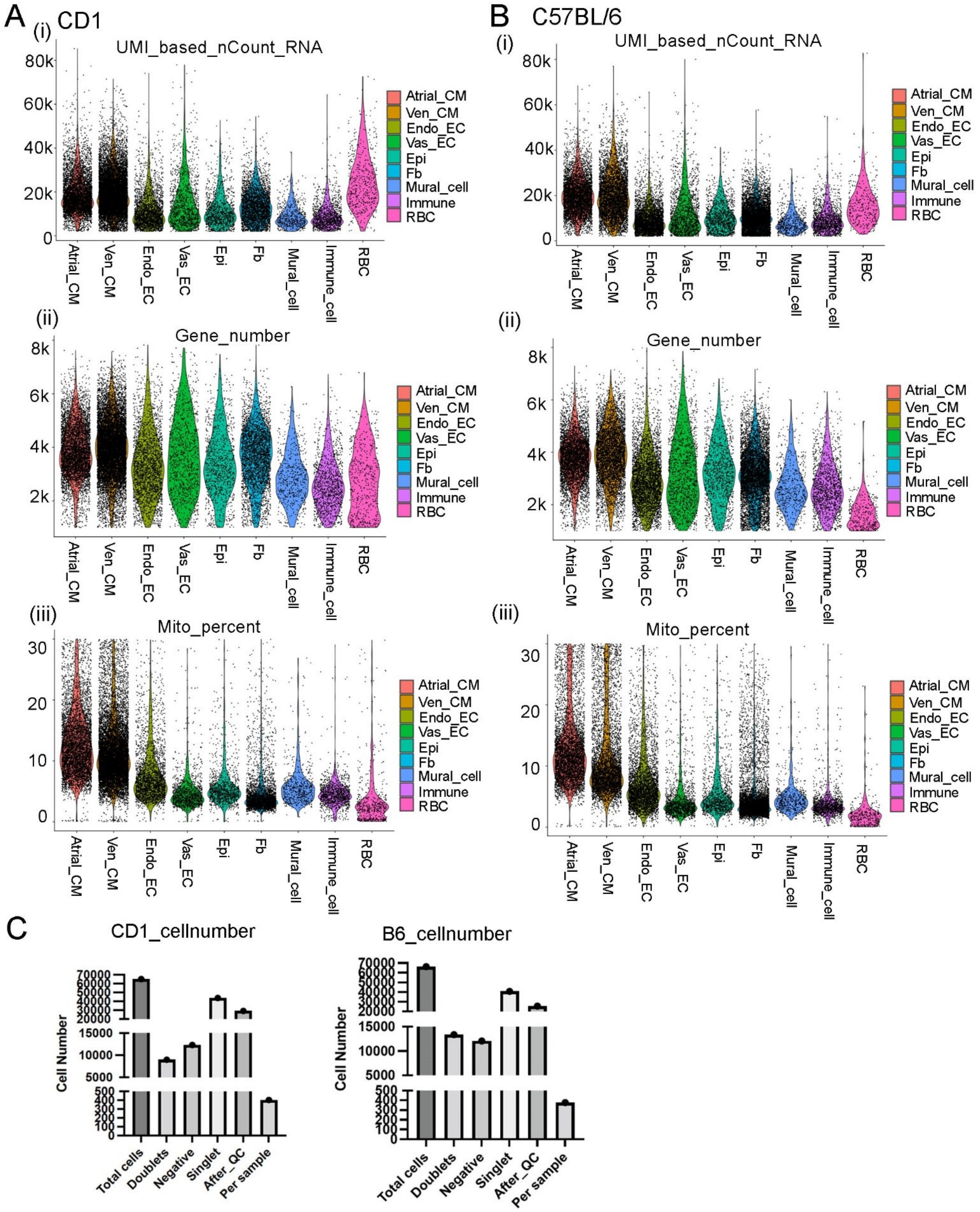

Fig S6. Quantitative analysis of the cells in CD1 and C57BL/6 datasets. (A, B) The number of sequencing reads, gene numbers, and percentage of mitochondria genes at each cell type in CD1 and C57BL/6. (C, D) The number of cells at different QC steps in CD1 and C57BL/6 datasets.

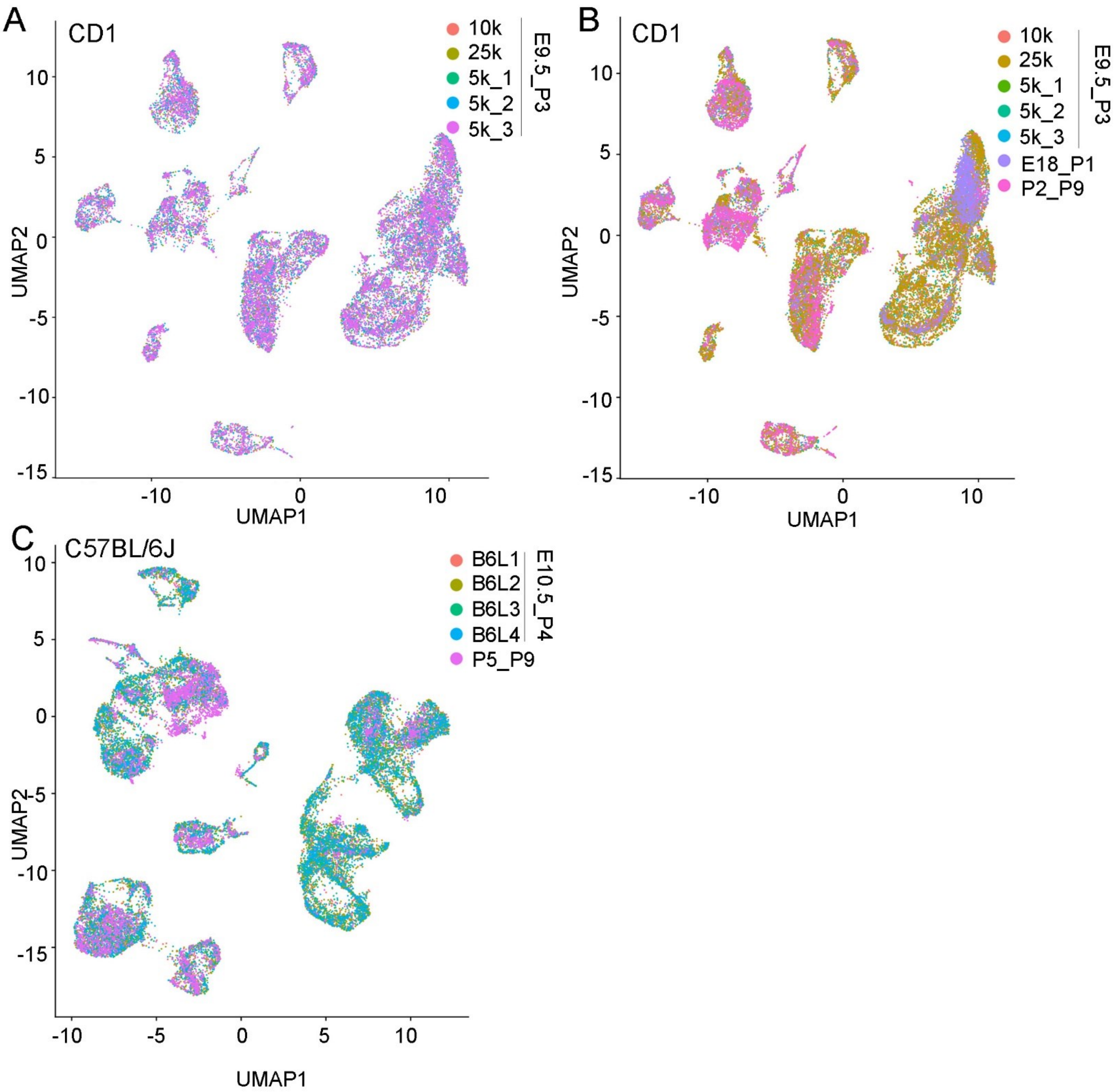

Fig S7. Integrative analysis of the single cells from different experiments. (A) The CD1 E9.5_P3 single cells were from five different loading wells with different targeting cell numbers. (B) UMAP plot of the CD1 data from different experiments (E9.5_P3, E18_P1, P2_P9). (C) The C57BL/6 single cell data profiled at different experiments. The UMAP plots were labeled by loading wells or experiments.

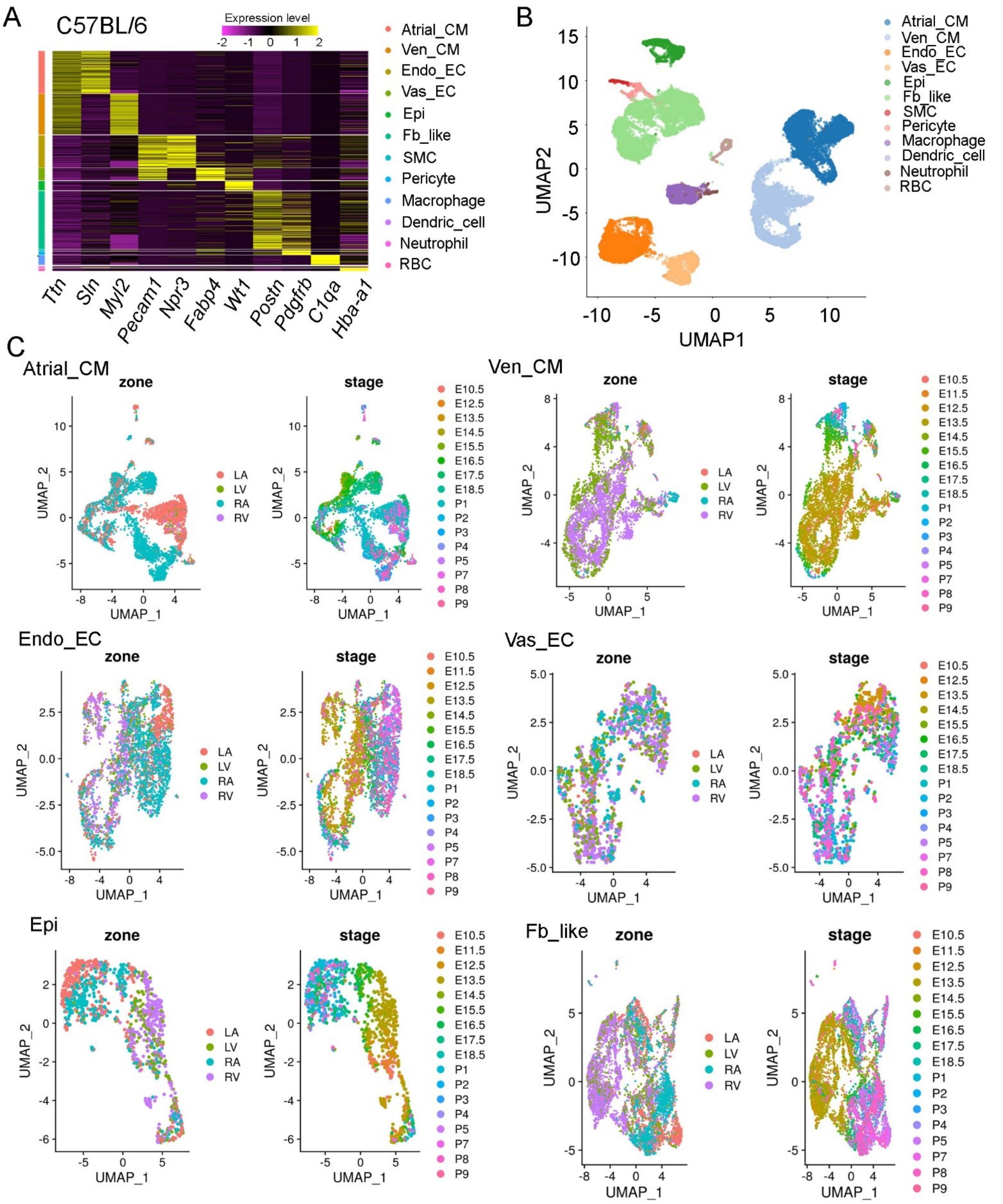

Fig S8. Analysis of the C57BL/6 single cell data. (A) Heatmap showing the expression pattern of cardiac lineage genes. (B) UMAP plot of the single cells labeled by cell types. (C) Zone and stage analysis of the cells in each cell type.

A

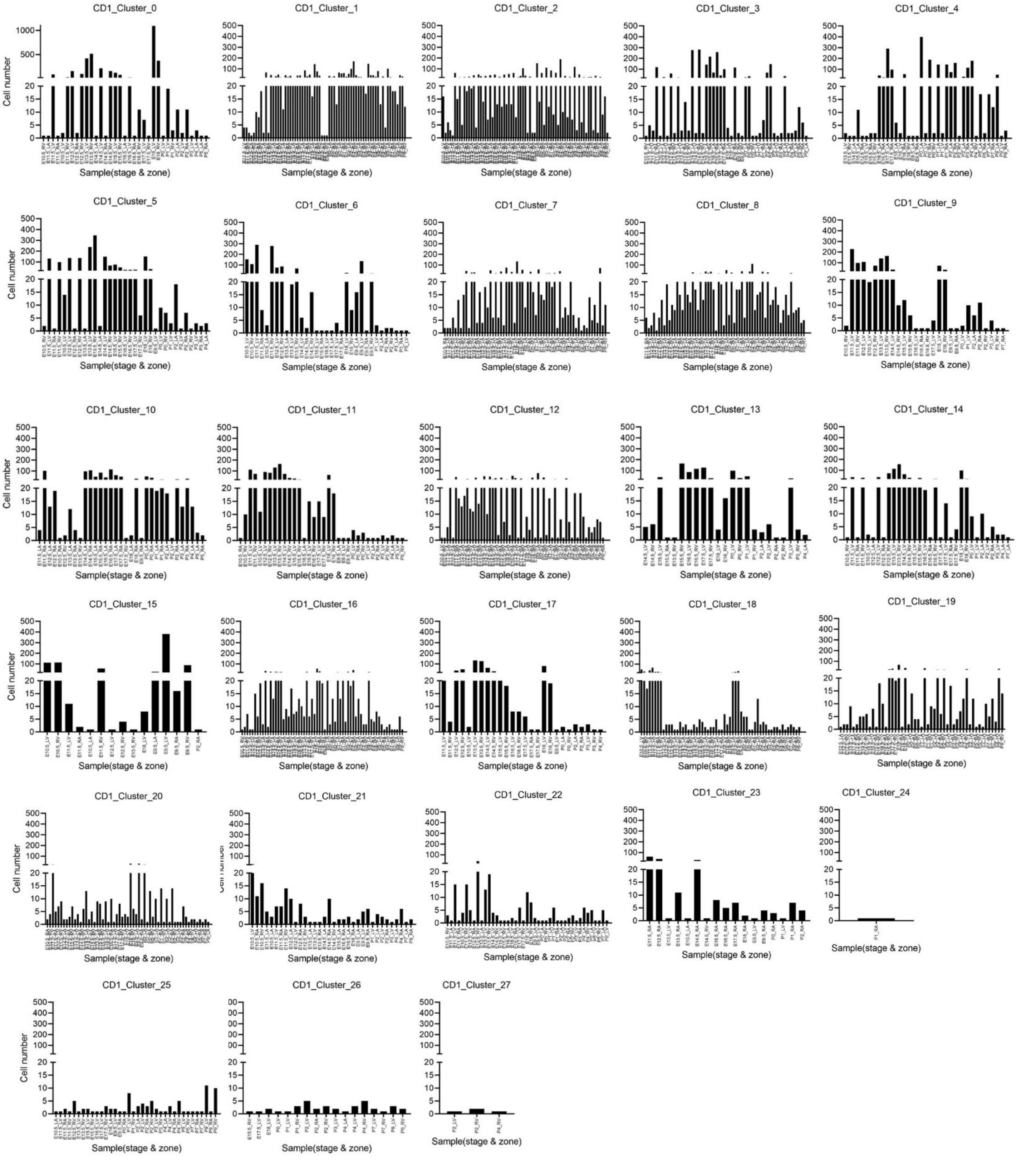

Fig S9. The number of cells in each sample at each CD1 cluster.

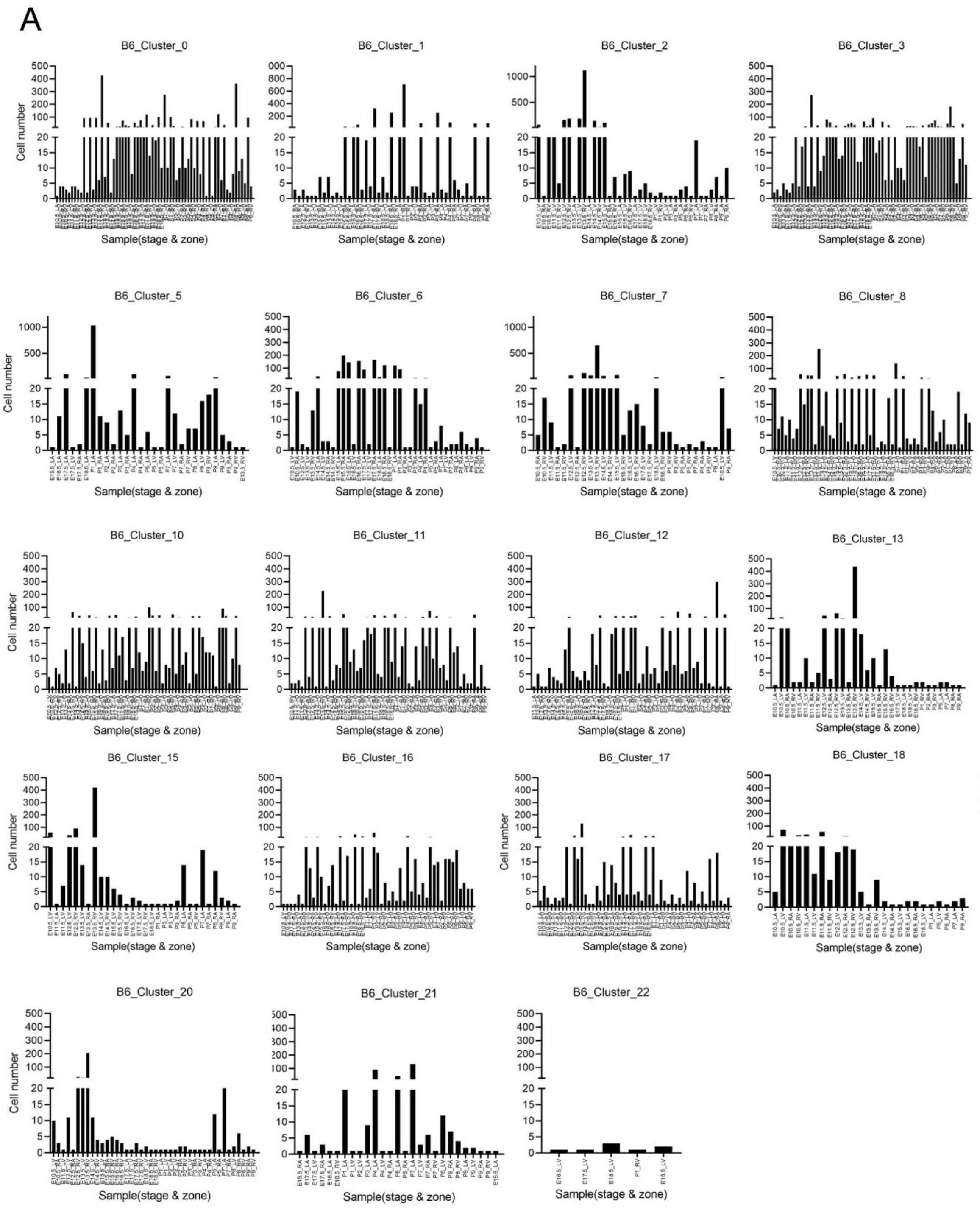

Fig S10. The number of cells in each sample at each C57BL/6 cluster.

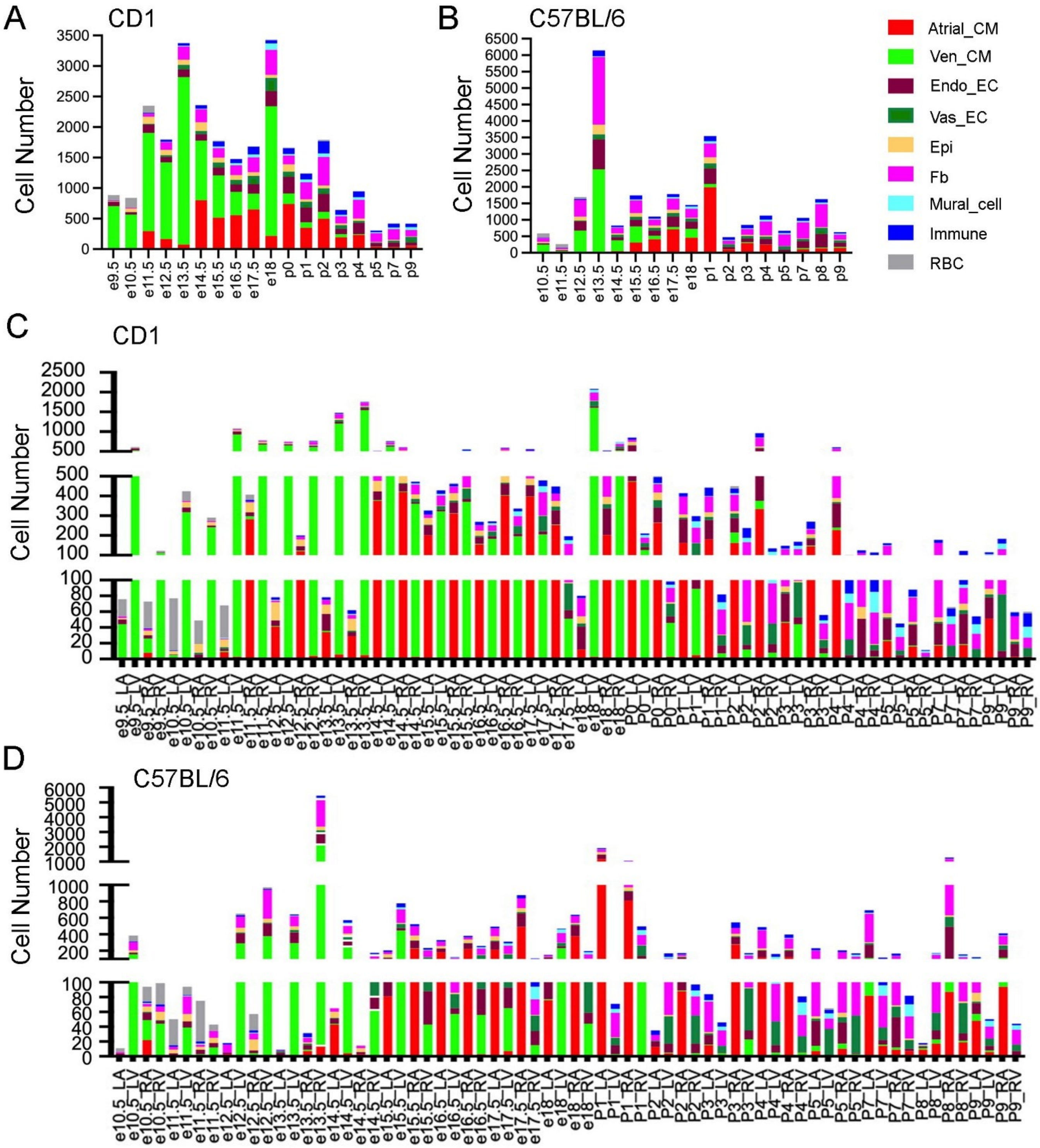

Fig S11. The cell type compositions at each stage and sample. (A, B) The cell type compositions at each stage in CD1 and C57BL/6 datasets. (C, D) The cell type compositions in each sample in CD1 and C57BL/6 datasets.

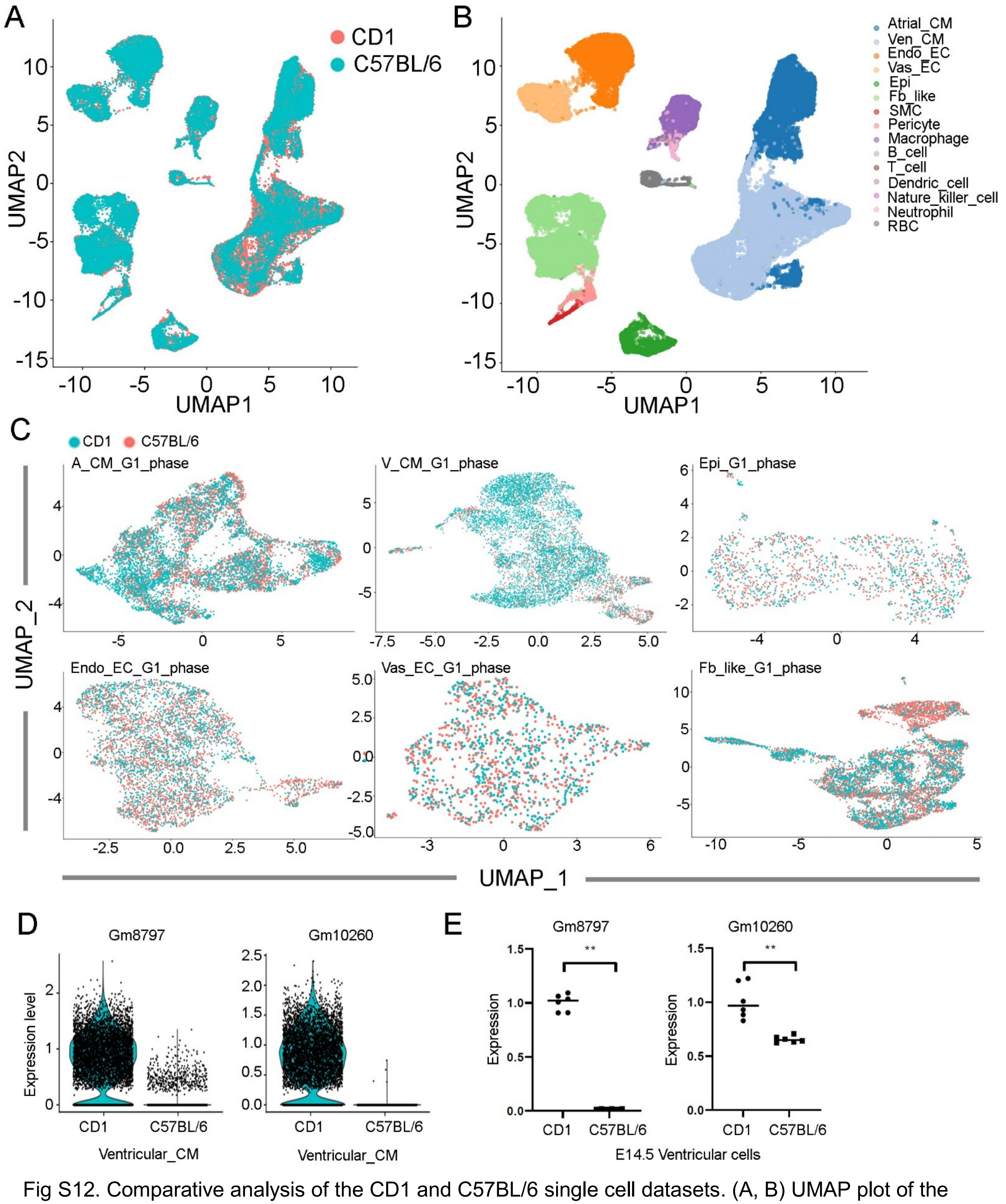

Fig S12. Comparative analysis of the CD1 and C57BL/6 single cell datasets. (A, B) UMAP plot of the integrated CD1 and C57BL/6 datasets labeled by mouse strain or cell type. (C) UMAP plot of the integrated G1 phased cells in each cell type. (D) The two representative genes differentially expressing in CD1 and C57BL/6 ventricular _CMs. (E) qPCR analysis confirmed the differential expression of the two genes between strains. N=2 biologically independent experiments with 3 replicates in each experiment. Student's t-test with two-tailed distribution was used for the statistical analysis. ** indicates the significance with p value <0.01.

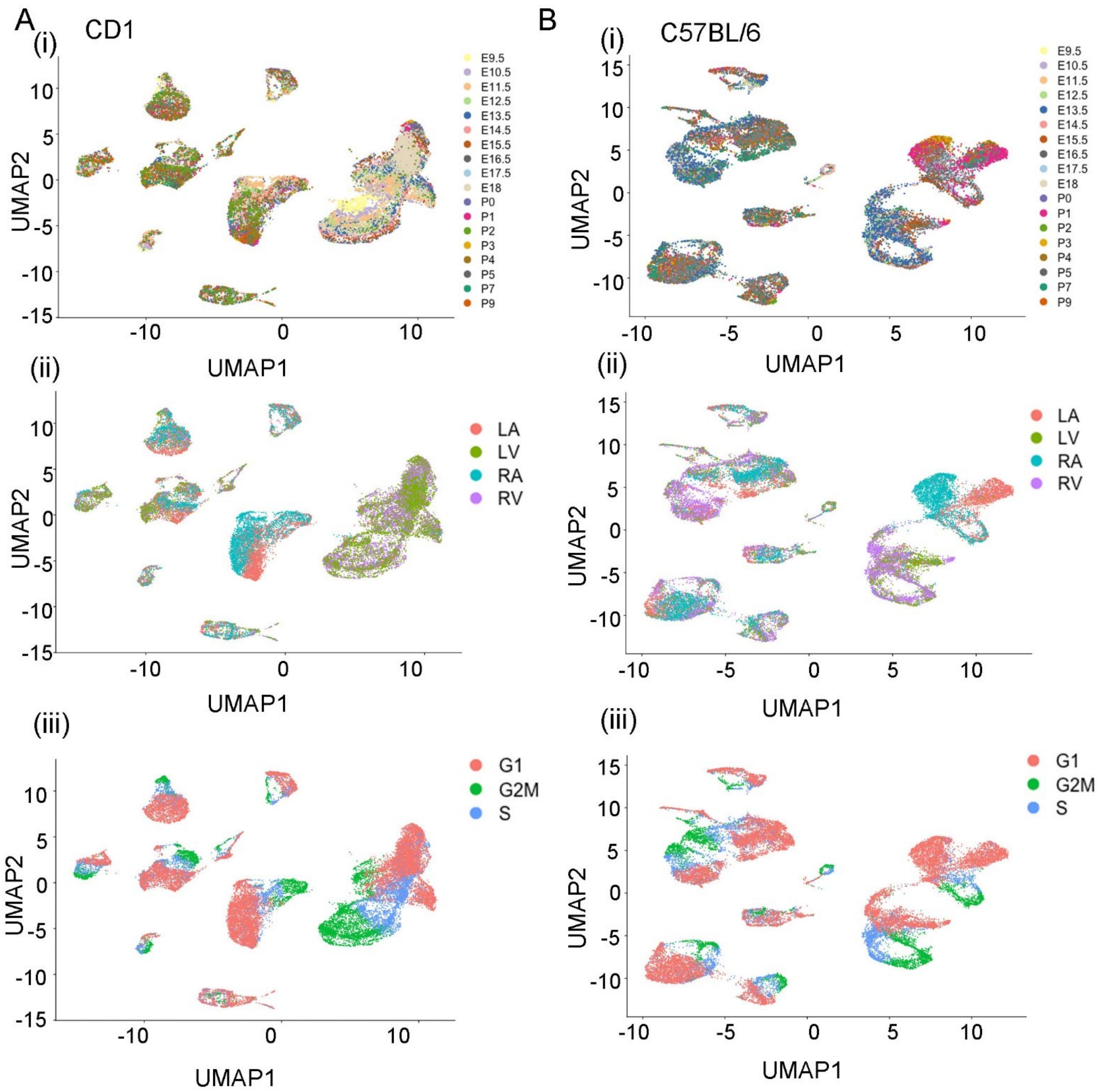

Fig S13. UMAP plots of the CD1 and C57BL/6 single cells with different labels. (A, B) The single cells from CD1 and C57BL/6 strains were labeled by stage, zone, or cell cycle phase.

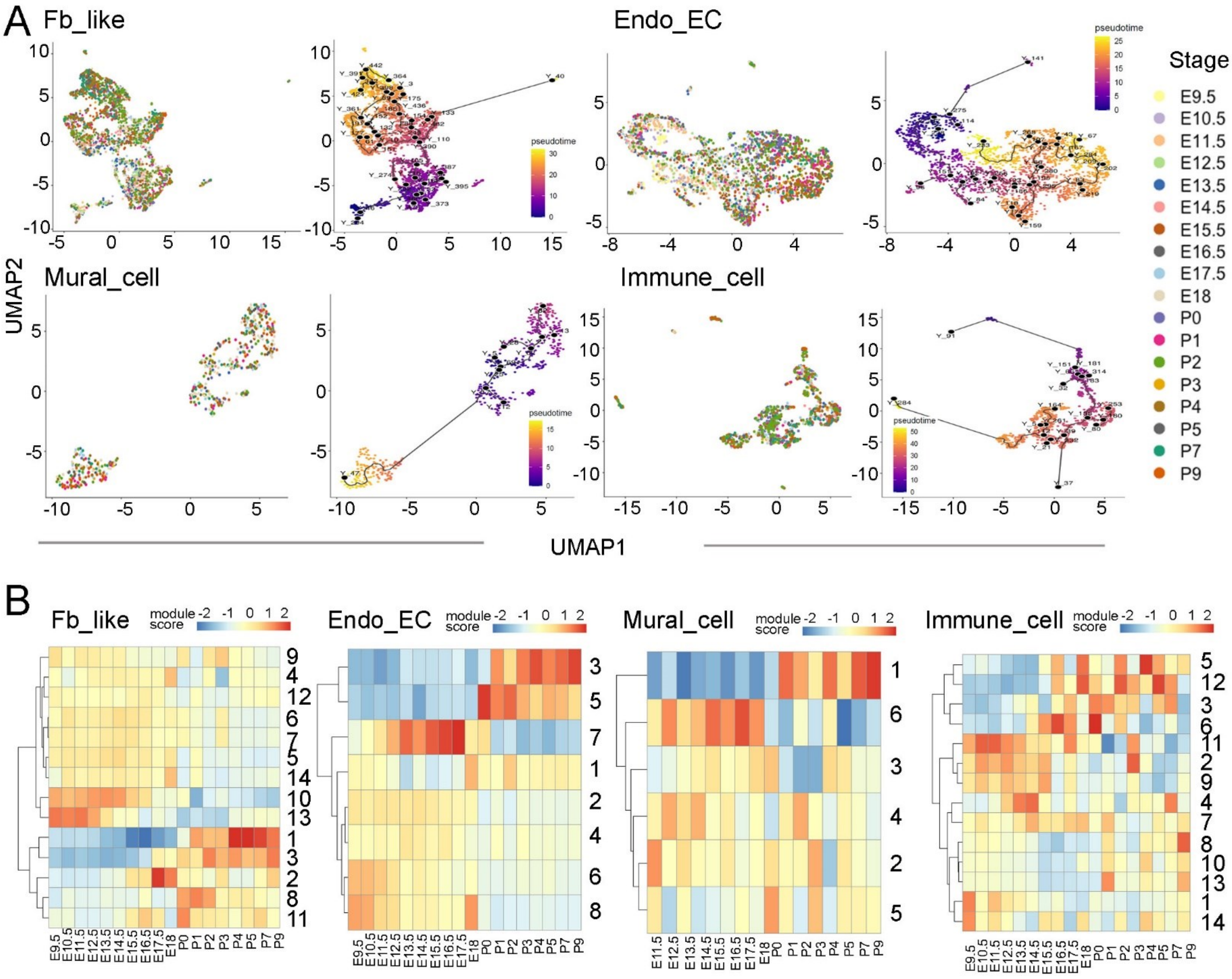

Fig S14. Stage analysis of the single cells in Fb_like, Endo_EC, Mural cell, and Immune cell. (A) UMAP plot of the single cells labeled by stage or pseudotime. (B) Identification of gene modules in each cell type. The color represents module score.

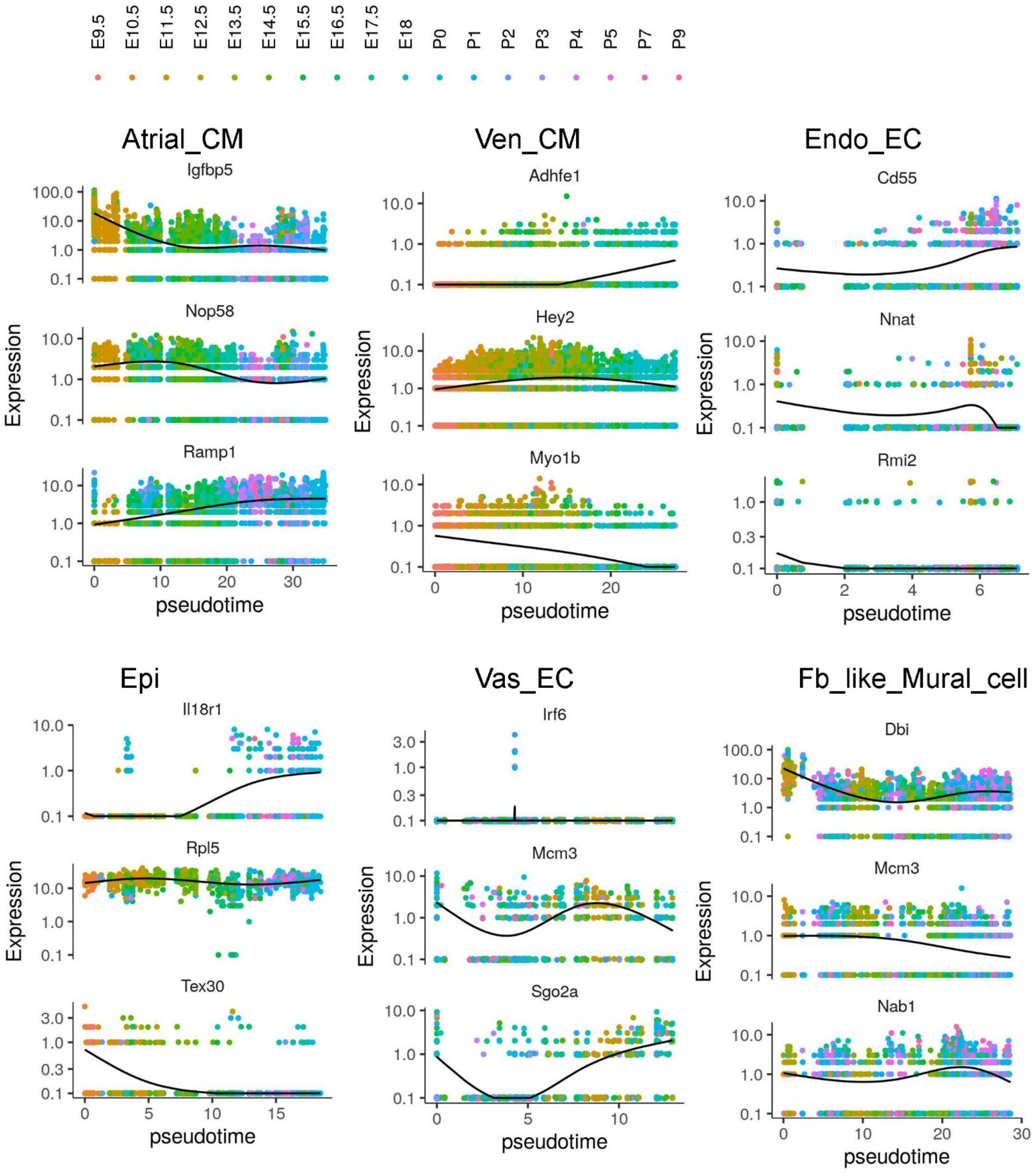

Fig S15. Representative genes that display pseudotime stage-specific expression pattern in each cell type. The color represents real stages. The cells were ordered along the x-axis by pseudotime, and the Y-axis represents gene expression level.

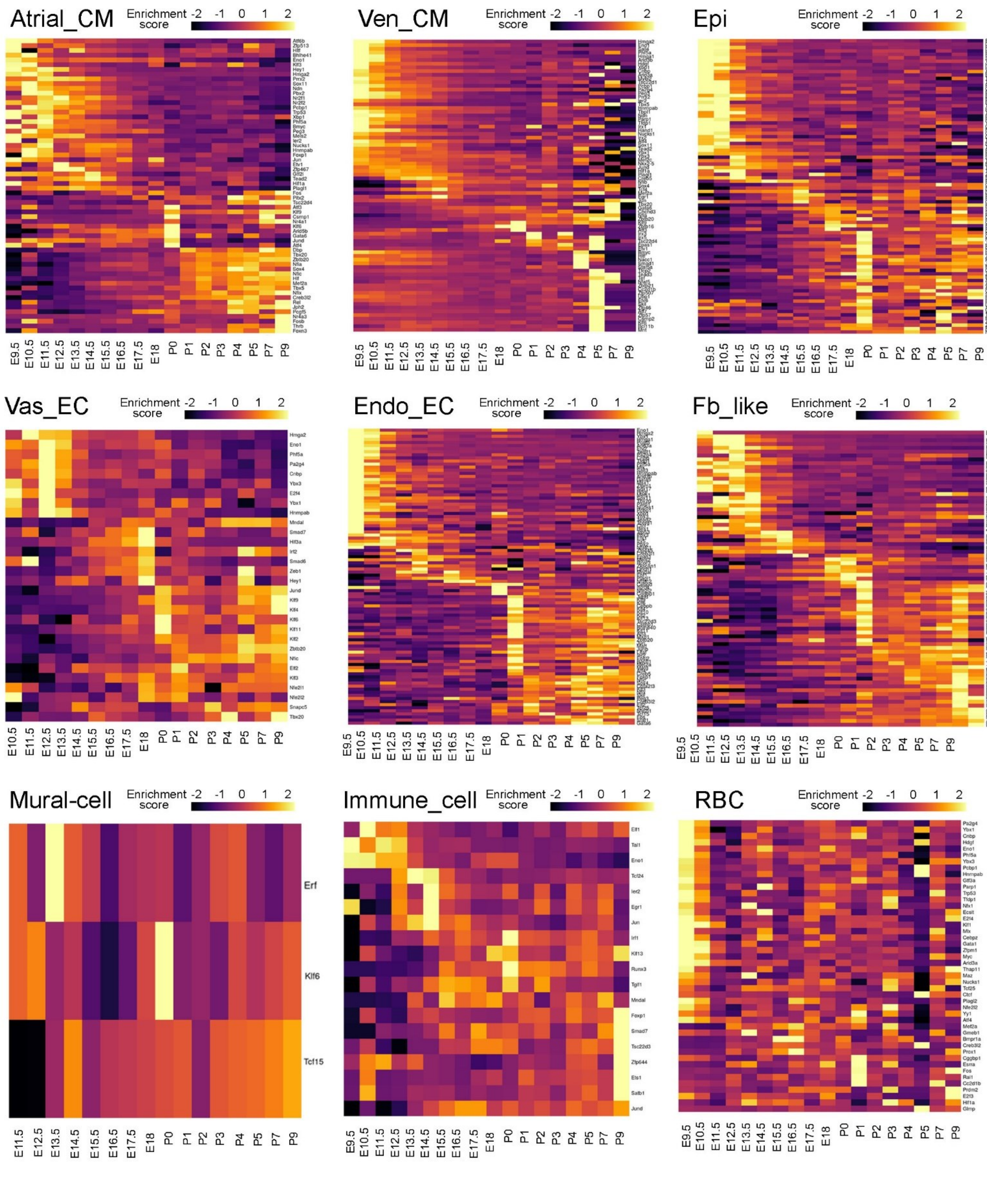

Fig S16. The stage specifically expressed transcription factors in each cell type. The gene expression level reduced from yellow to blue to black.

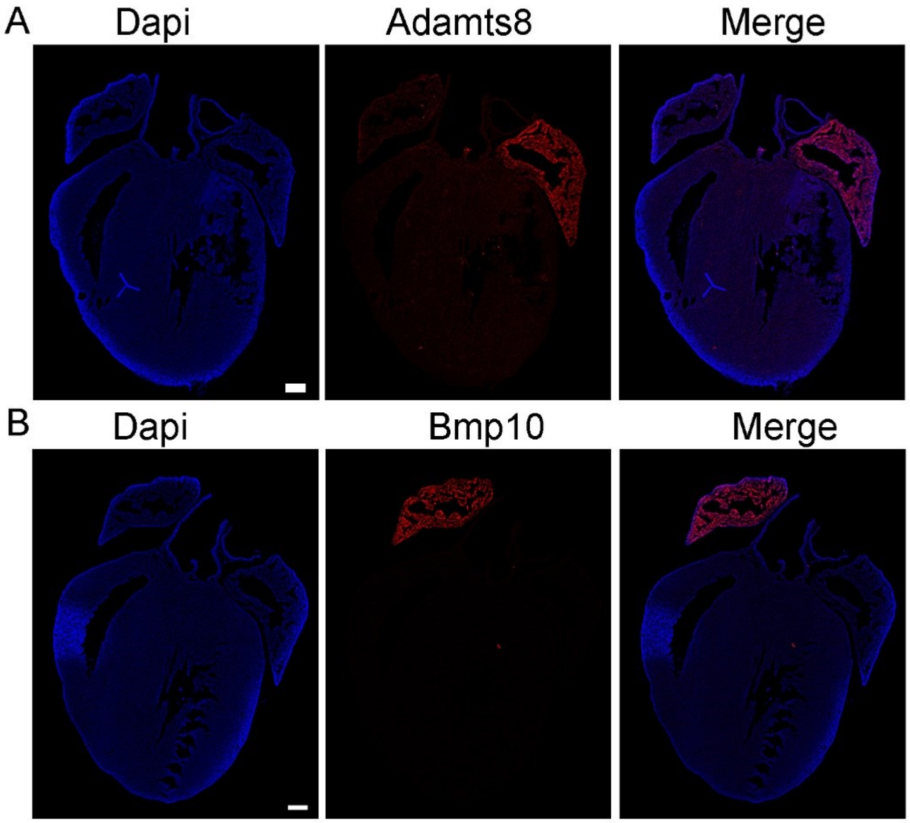

Fig S17. Staining analysis of *Adamts8* and *Bmp10*. (A, B) mRNA staining showed that *Adamts8* and *Bmp10* were specifically expressed in LA and RA at P3 hearts, respectively. The staining experiments were repeated twice with similar results. Scale bar=500 μm.

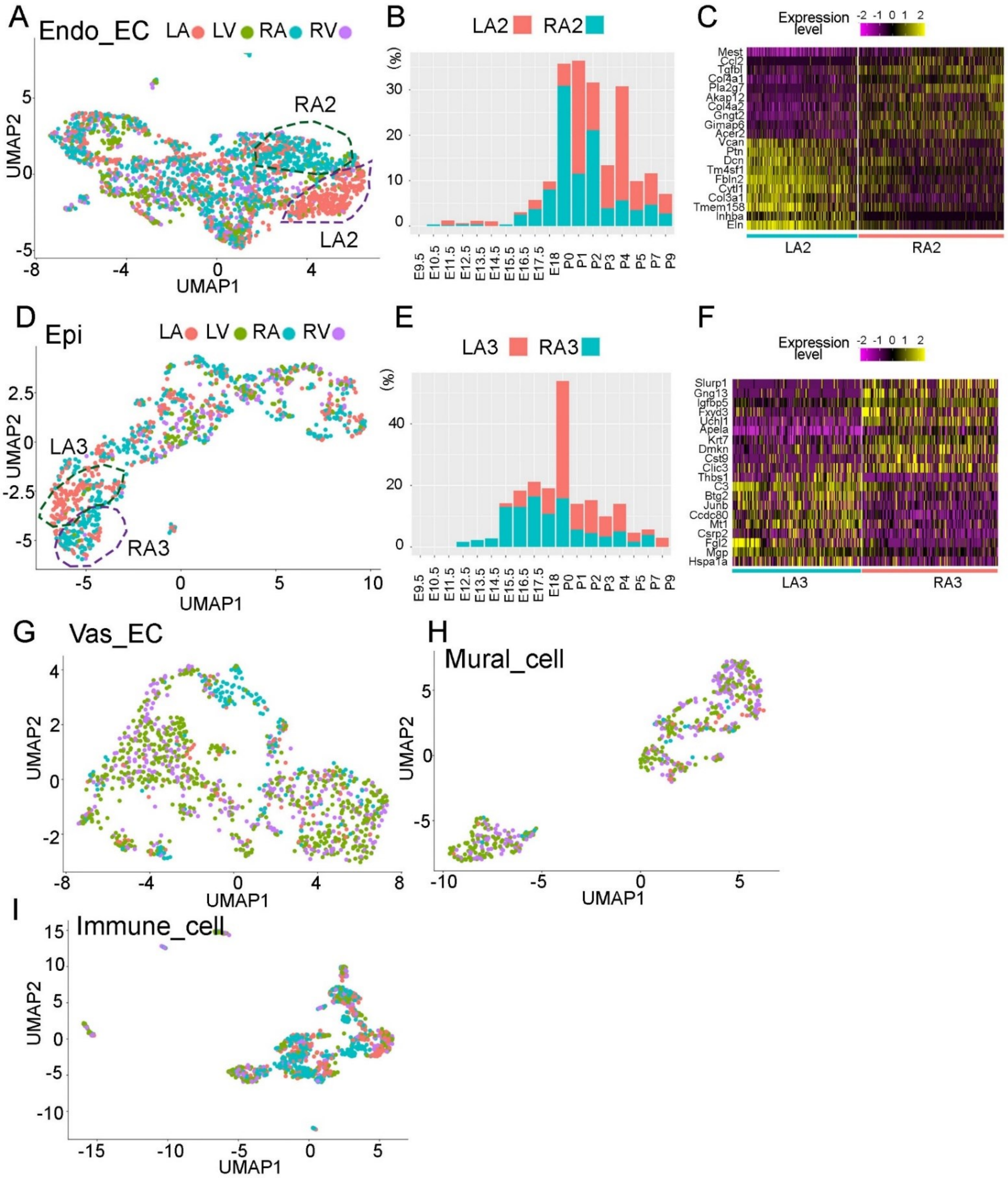

Fig S18. The zone-specific molecular signatures in five cell types. (A) UMAP plot of Endo_ECs labeled by zones. One LA and one RA-specific cell populations (LA2, RA2) were identified. (B) Stage analysis of the cells in LA2 and RA2. (C) Expression heatmap of the top 20 genes that were differentially expressed in LA2 and RA2 cells. (D) UMAP plot of epicardial cells labeled by zones. One LA and one RA-specific cell populations (LA3, RA3) were identified. (E) Stage analysis of the cells in LA3 and RA3. (F) Expression heatmap of the top 20 genes that were differentially expressed in LA3 and RA3 cells. (G-I) UMAP plots of Vas_EC, Mural_cells, and Immune cells labeled by zones.

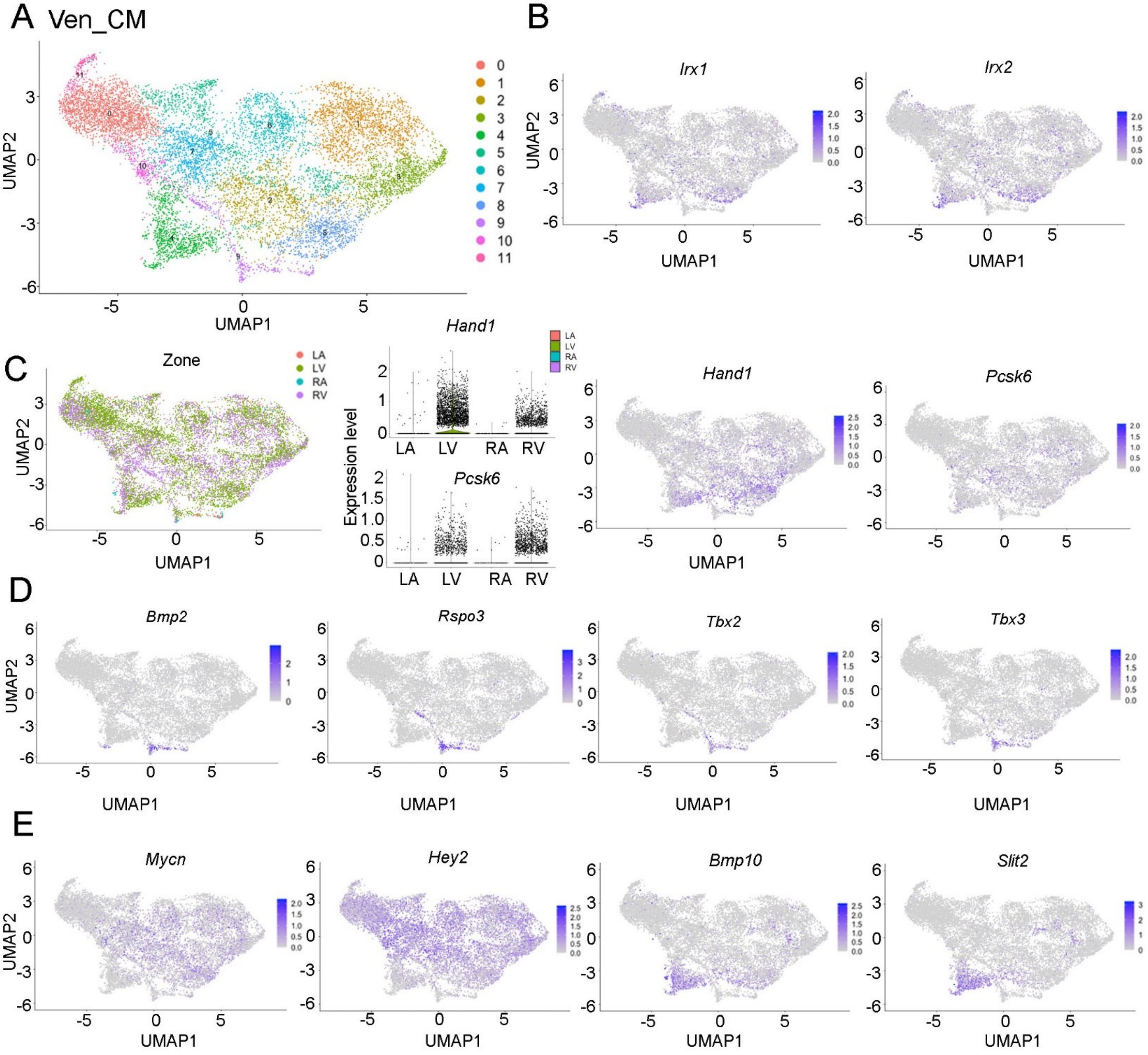

Fig S19. The cellular heterogeneity in Ven_CM. (A) UMAP plot of Ven_CM labeled by clusters. (B) *Irx1* and *Irx2* positive CMs were distributed in multiple cell clusters. (C) UMAP plot of Ven_CMs labeled by zone. No LV and RV-specific clusters were identified. Violin plots showed a preferential expression of *Hand1* and *Pcsk6* in LV and RV CMs, respectively. The feature plots did not identify cluster-specific expressions of *Hand1* and *Pcsk6*. (D) The cluster 9 CMs highly expressed AVC marker genes *Bmp2*, *Rspo3*, *Tbx2*, and *Tbx3*. (E) The cluster 4 CMs highly specifically expressed trabecular myocardium genes *Bmp10* and *Slit2*, and the cells in other clusters expressed compact myocardium genes *Mycn* and *Hey2*.

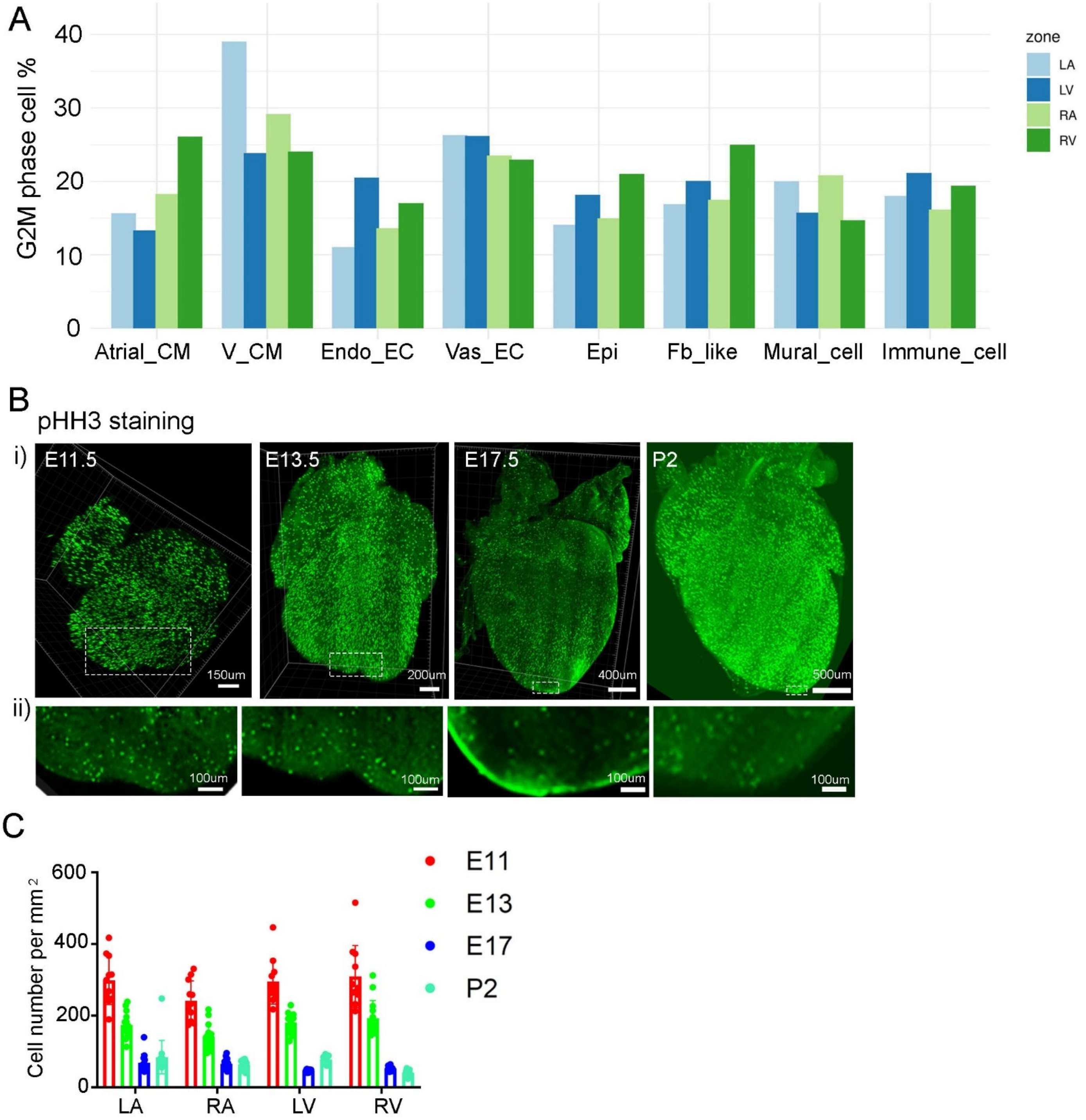

Fig S20. The proportion of cells in proliferation at different stages and zones. (A)The percentages of G2M phased cells in each cell type at each chamber. (B) (i) Overview and (ii) enlarged portion of cleared hearts with pHH3 staining at four stages. (C) The pHH3 positive cells declined along developmental progression in all four chambers. iDISCO images scale bar: E11.5 = 150 μm; E13.5 = 200 μm; E17.5 = 400 μm; P2 = 500 μm. N= around 10 section images from one cleared heart at each stage were analyzed. The error bars represent SD. ANOVA with Tukey's multiple comparisons were used for the statistical analysis. All comparisons except the E17 and P2 pair are statistical significant with p value<0.01.

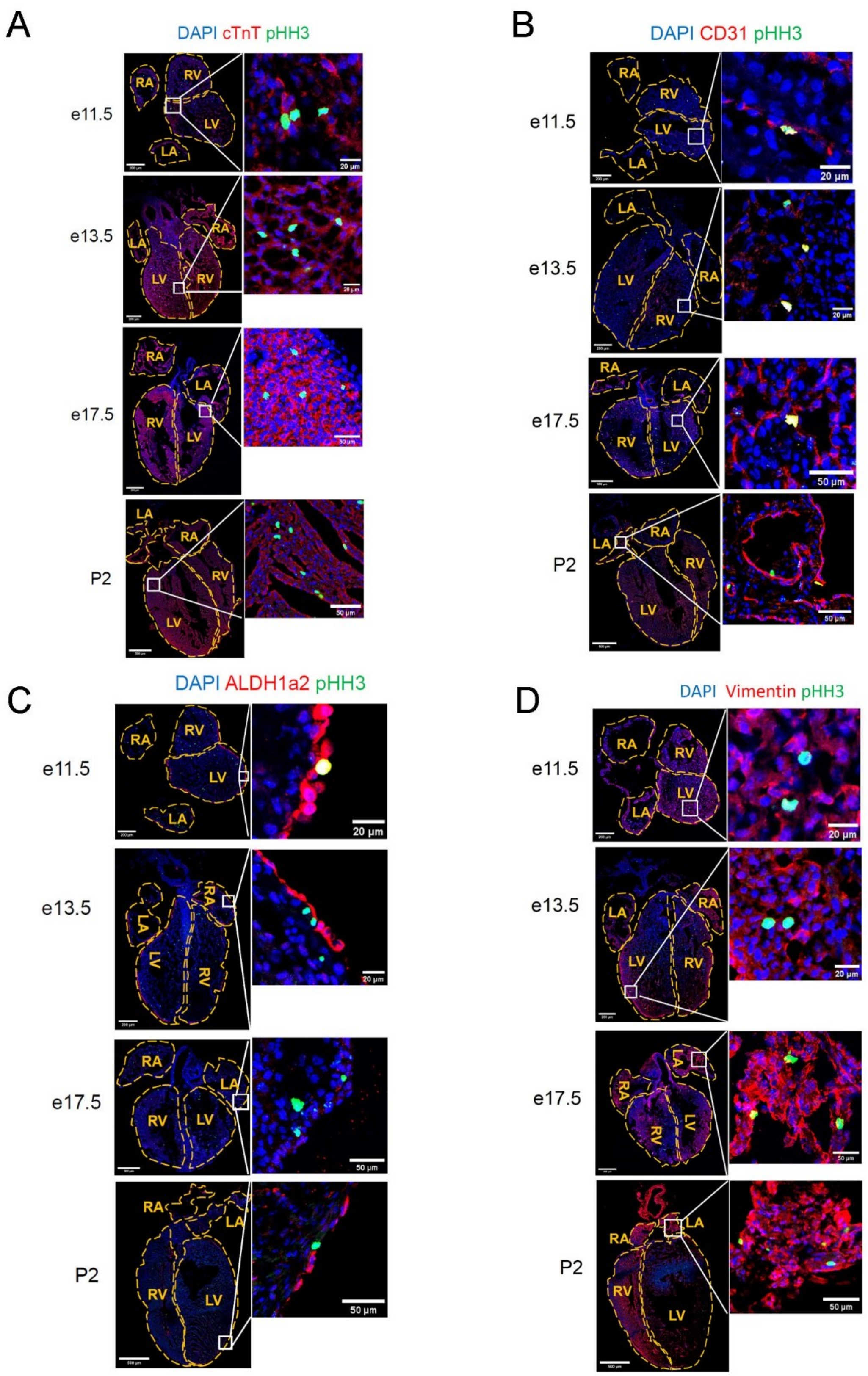

Fig S21. Representative images staining for pHH3 together with (A) CM lineage gene cTNT, (B) endothelial cell gene CD31, (C) epicardial cell gene ALDH1A2, and (D) fibroblast gene VIM. Scale bar=200µm in the images with whole heart sections at E11.5 and E13.5. Scale bar=500µm in the images with whole heart sections at E17.5 and P2. Scale bar=20µm in the enlarged images at E11.5 and E13.5. Scale bar=50µm in the E17.5 and P2 enlarged images. The staining experiments were repeated on three heart sections with similar results.

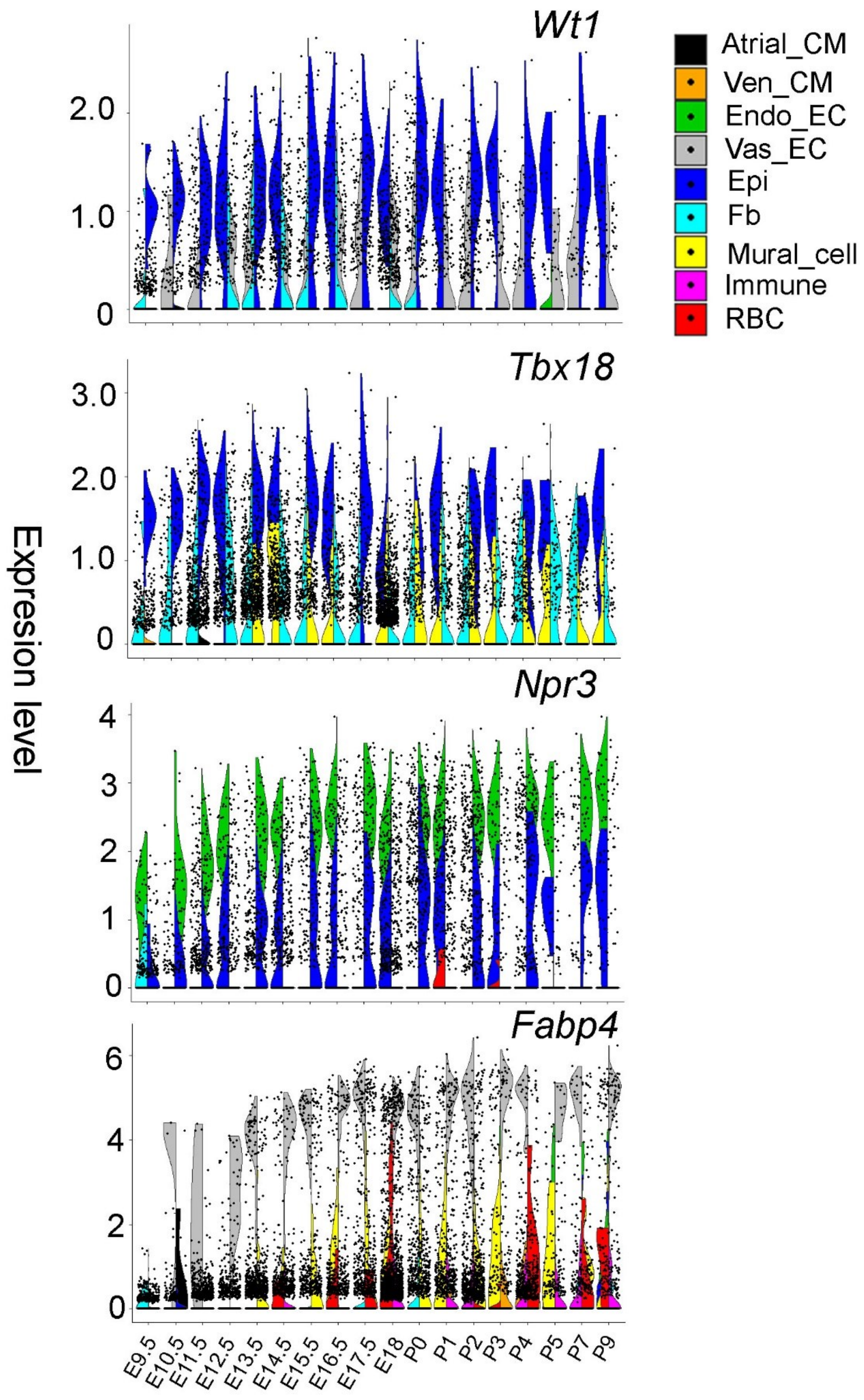

Fig S22. Violin plots showing the expression of genes *Wt1*, *Tbx18*, *Npr3*, and *Fabp4* in each cell type across the stages.

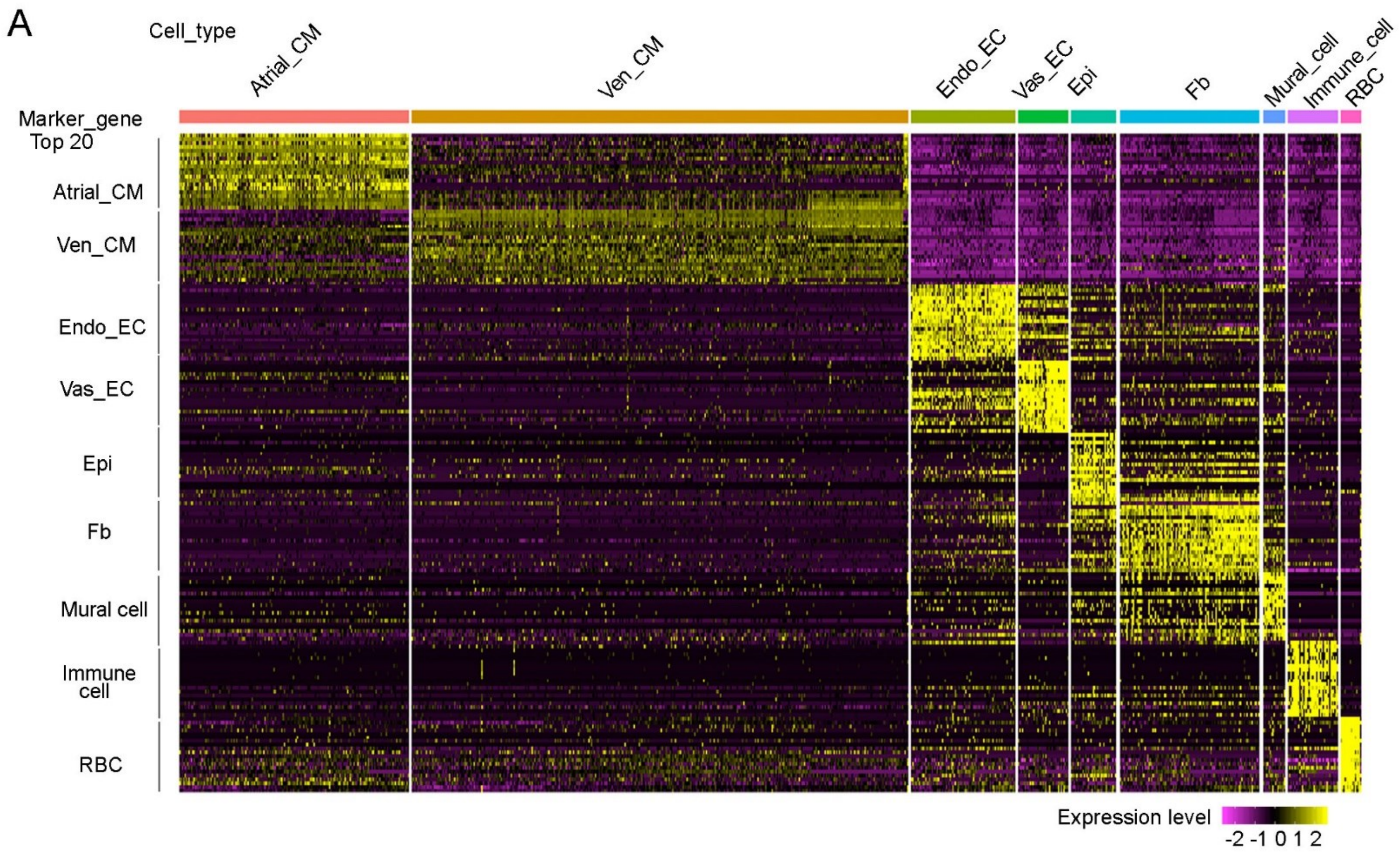

Fig S23. Expression heatmap of the top 20 genes that were uniquely expressed in each cell type.

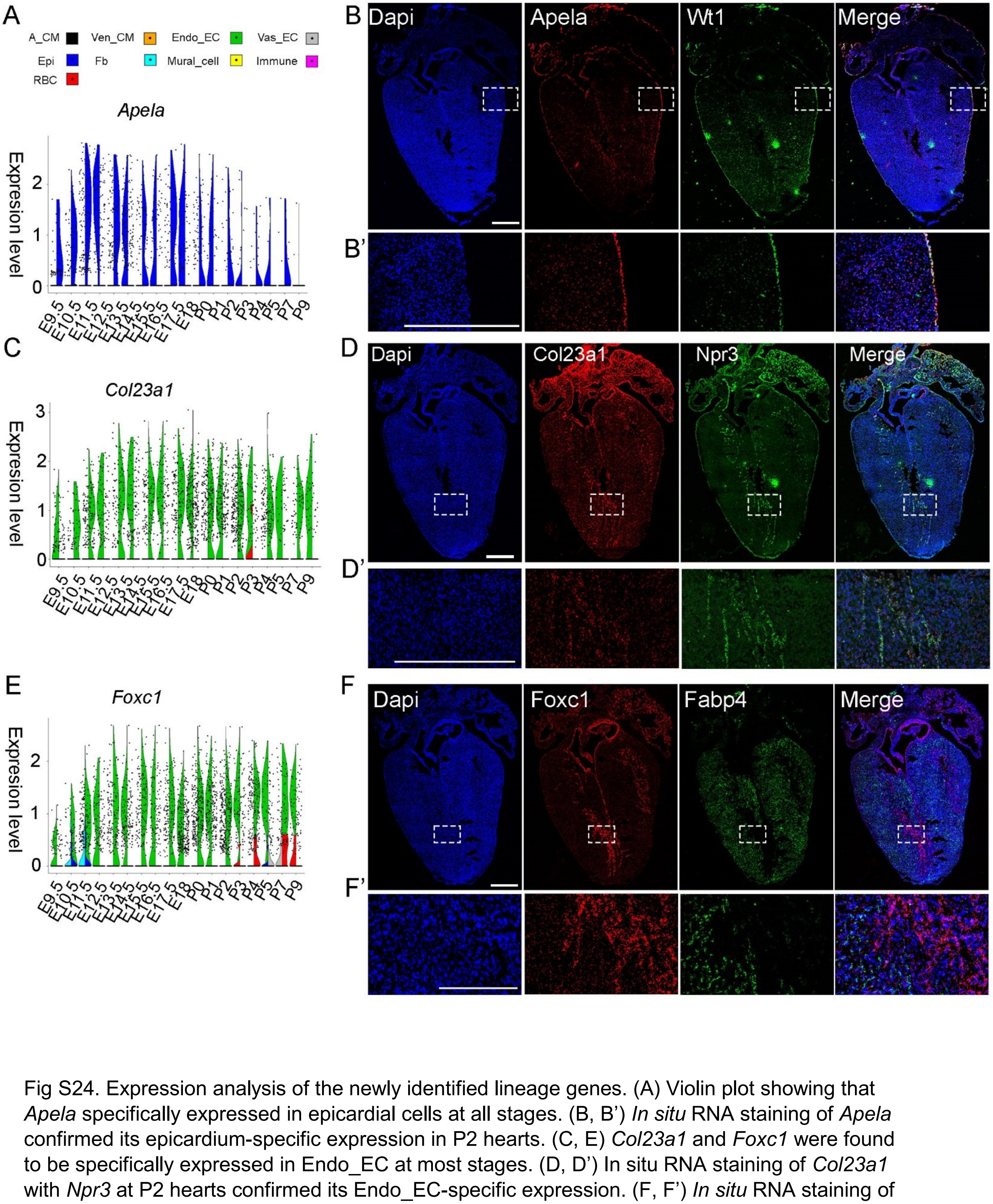

Fig S24. Expression analysis of the newly identified lineage genes. (A) Violin plot showing that *Apela* specifically expressed in epicardial cells at all stages. (B, B') *In situ* RNA staining of *Apela* confirmed its epicardium-specific expression in P2 hearts. (C, E) *Col23a1* and *Foxc1* were found to be specifically expressed in Endo_EC at most stages. (D, D') In situ RNA staining of *Col23a1* with *Npr3* at P2 hearts confirmed its Endo_EC-specific expression. (F, F') *In situ* RNA staining of *Foxc1* and *Fabp4* confirmed their complement expression pattern, indicating *Foxc1* expresses in Endo_EC. The staining experiments were repeated twice with similar results. Scale bar=500 μm.

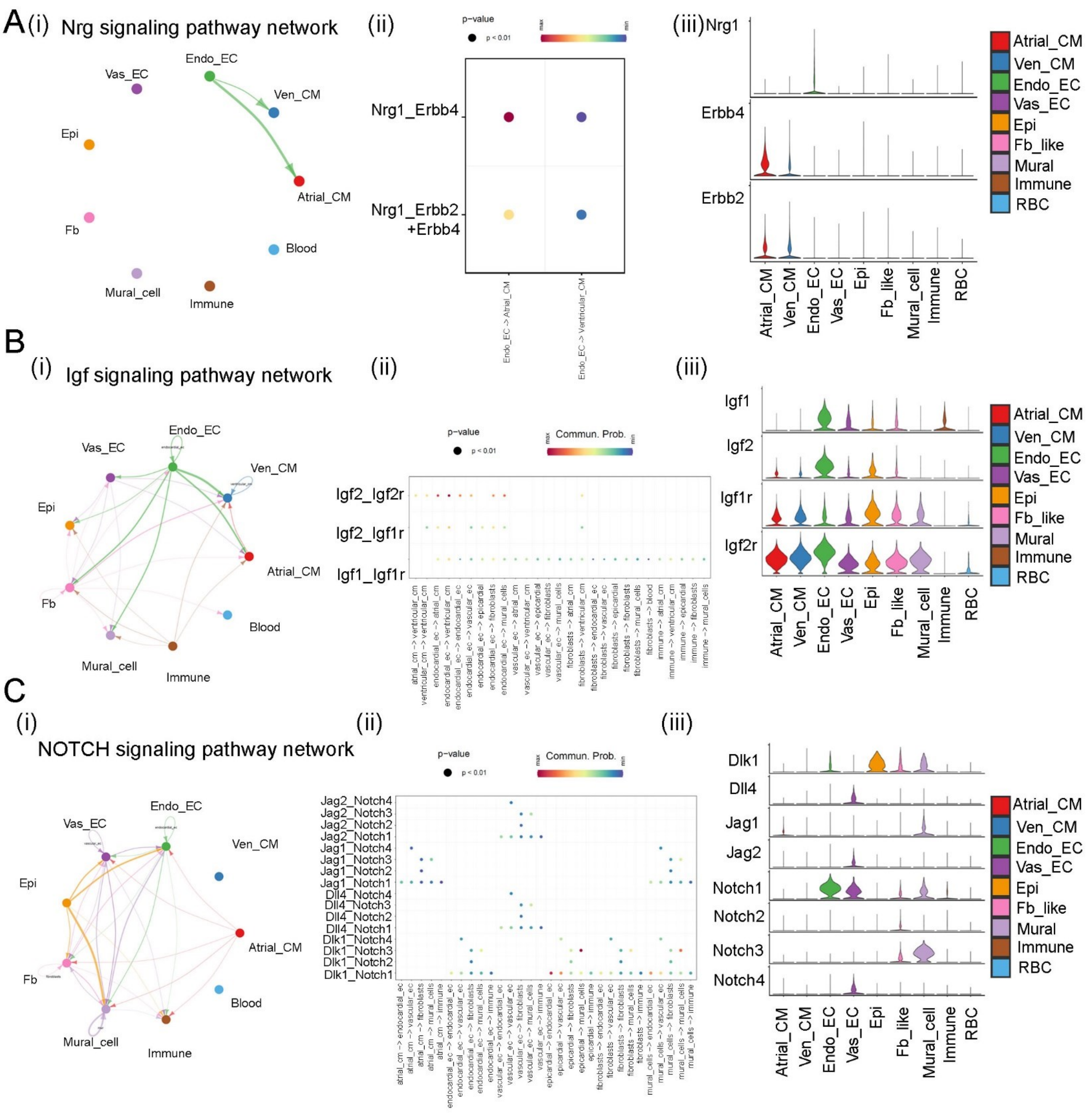

Fig S25. The ligand-receptor interactions in three representative signaling pathways. (A)(i) The network of Nrg signaling pathway. The Loops outside represent autocrine signals for each cell type; the lines connecting cell types represent paracrine signals. The lines with the same color as the cell type dots indicate the cell type secreted ligand in the interactions; the line thickness correlates with the amount of interactions. (ii) The specific ligand-receptor interactions. Each dot represents interactions between the pair of cell types. The color represents interaction probabilities. (iii) Violin plots showing the expression of ligands and receptors at each cell type. (B-C) The signaling pathway networks, ligand-receptor interactions, and ligand and receptor expression patterns in Igf and Notch signaling pathways.

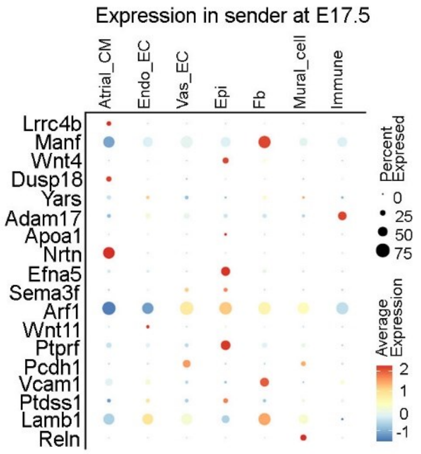

Fig S26. The expression pattern of epicardial cell derived ligands that potentially regulated genes expression in Ven_CMs at E17.5.

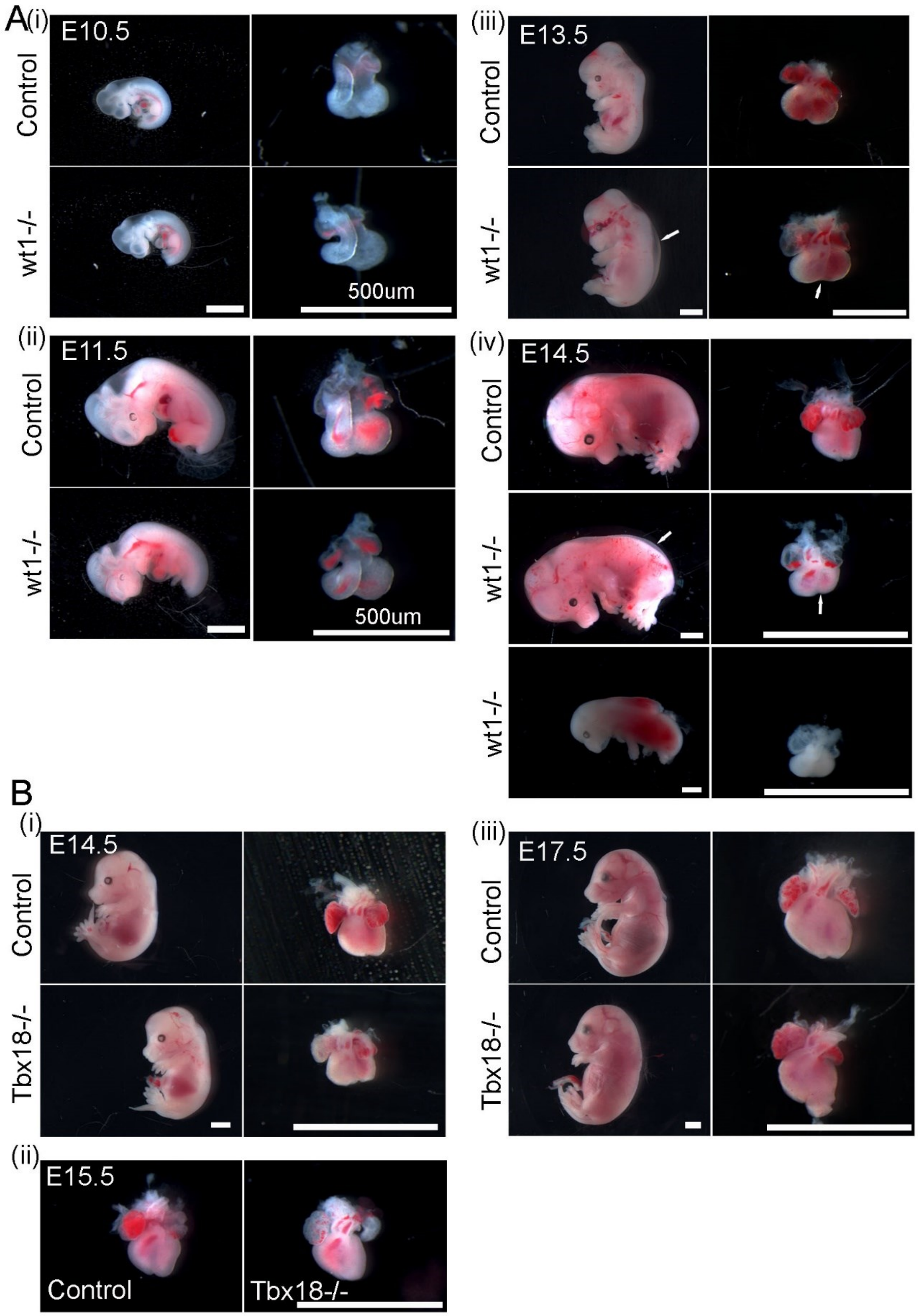

Fig S27. Representative images of control and *Wt1* or *Tbx18* mutant embryos and hearts. (A)(i-iv) Representative embryos and hearts of *Wt1* mutants and wildtype controls from the same litter at different stages. At E13.5 and E14.5, the *Wt1* mutant embryos have obvious body wall edema, and their hearts have more rounded and bifid apices, as pointed by the arrows. (B) (i-iii) Representative *Tbx18* mutant and control embryos and hearts at E14.5, E15.5, and E17.5. Except for the two labeled scale bars that represent 500um, all the other scale bars=1mm.

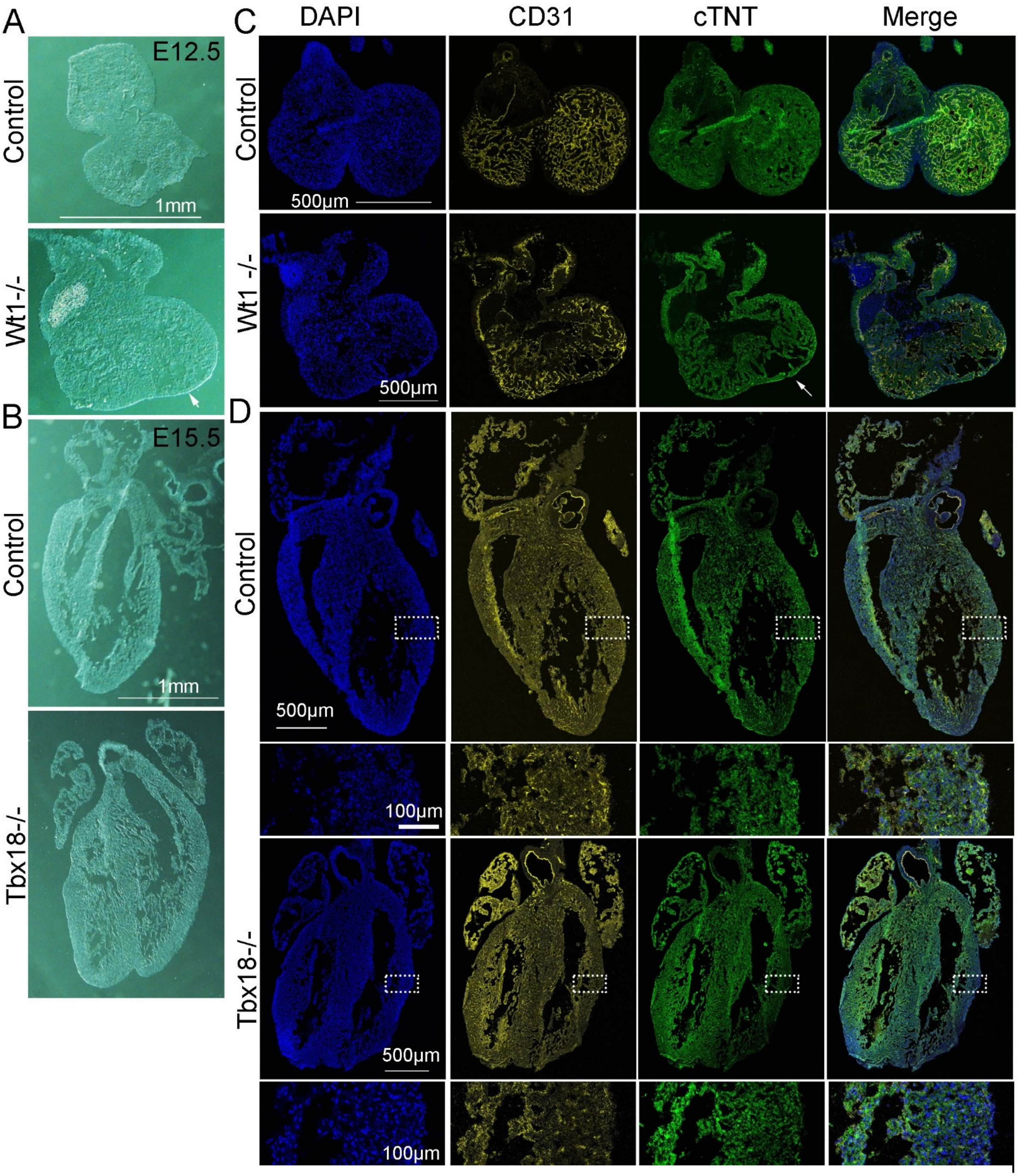

Fig S28. Histological and staining analysis of *Wt1* and *Tbx18* control and mutant hearts. (A, B) Histological images (cryo-sectioning) of *Wt1* control and mutant hearts at E12.5 and *Tbx18* control and mutant hearts at E15.5. (C) Staining analysis of CD31 (endothelial cell marker) and cTNT (cardiomyocyte marker) on control and Wt1 mutant heart sections at E12.5. Note that thinner myocardium was observed in *Wt1* mutant hearts than the control hearts (point by arrow). (D) Staining analysis of CD31 and cTNT on *Tbx18* control and mutant heart sections at E15.5. No obvious changes in vessel density were observed between controls and mutants. The staining experiments were repeated on three heart sections with similar results.

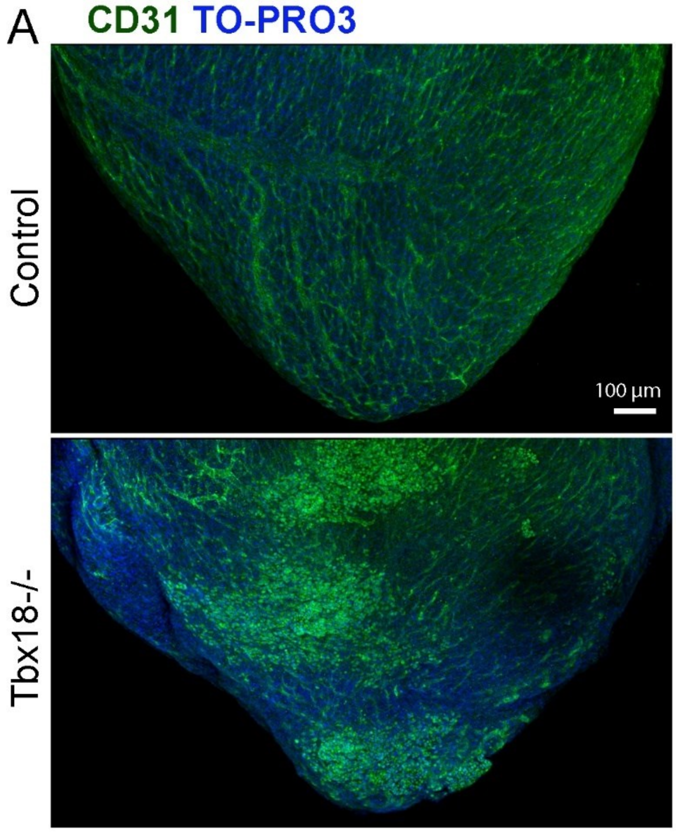

Fig S29. Whole mount staining analysis of CD31 in *Tbx18* control (*Tbx18*+/-) and mutant (*Tbx18*-/-) hearts at E17.5. Ectopic nodules with CD31-positive cells were observed in *Tbx18* mutant hearts. Only one mutant heart at this stage was analyzed, and its defects were observed. Scale bar=100 µm.

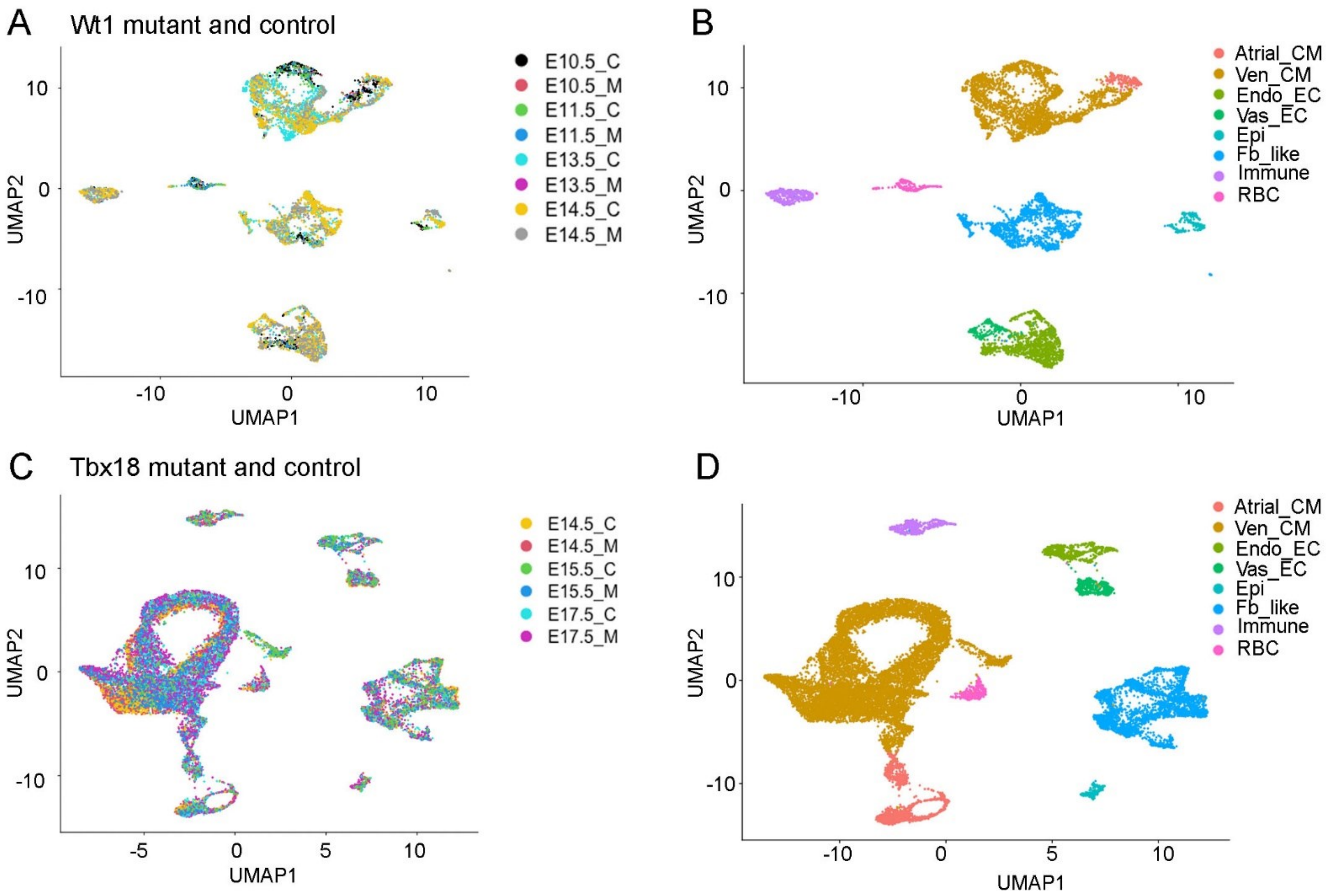

Fig S30. Unsupervised clustering analysis of the scRNA-seq data from *Wt1* and *Tbx18* mutant and control samples. (A, B) UMAP plots of *Wt1* mutant and control cells labeled by sample or cell type. (C, D) UMAP plots of *Tbx18* mutant and control cells labeled by sample or cell type.

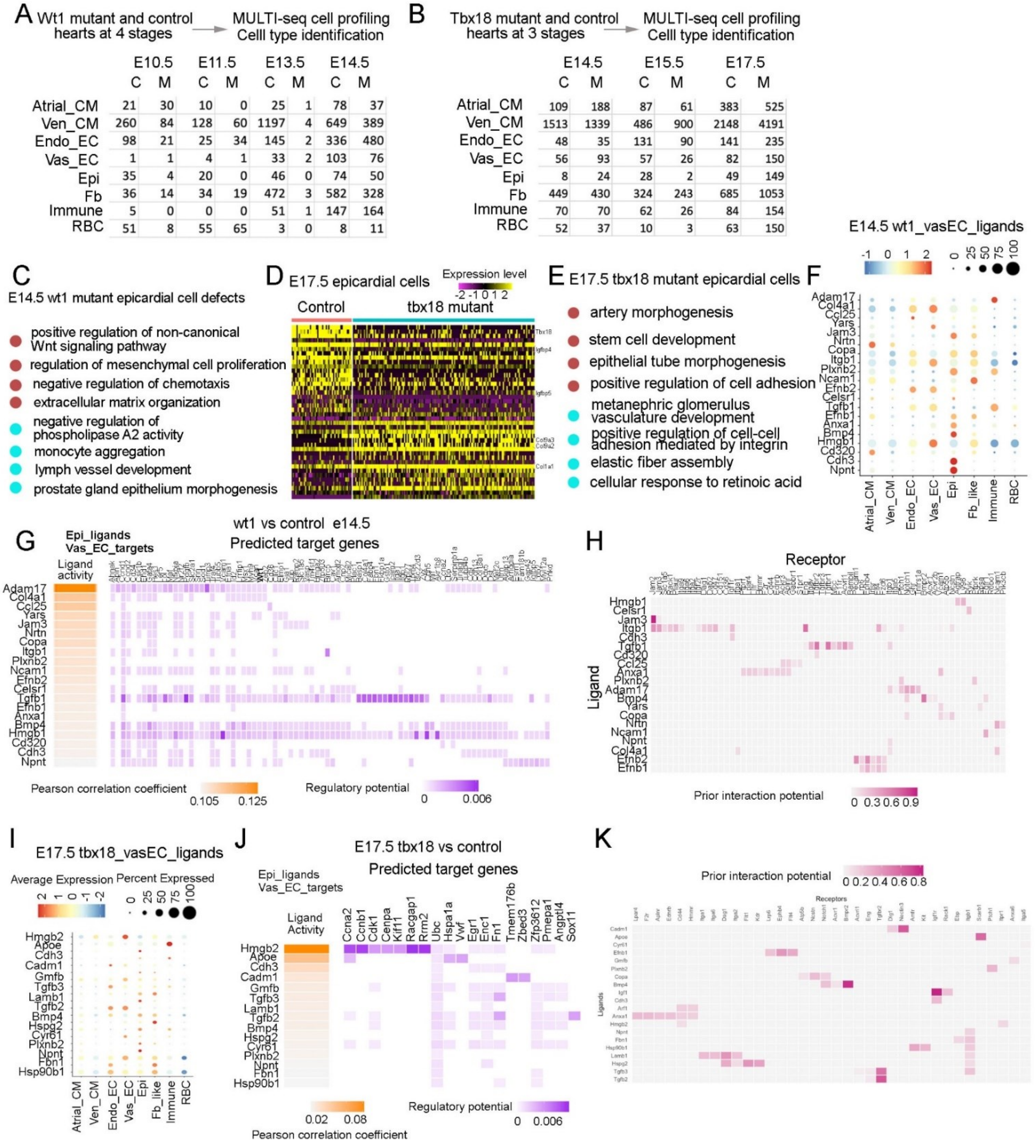

Fig S31. Detailed analysis of the *Wt1* and *Tbx18* mutant and control data. (A, B) The number of cells recovered in each cell type and sample after QC. (C) The gene pathways that are abnormally expressed in *Wt1* mutant epicardial cells at E14.5. (D, E) The expression heatmap and pathways of genes that differentially expressed in control and *Tbx18* mutant epicardial cells at E17.5. (F) The expression pattern of epicardial cell derived ligands that potentially regulated the gene's expression in Vas_ECs at e14.5 (analysis of *Wt1* mutant). (G, H) The activity of epicardial cell derived ligands, and their prior interaction potentials with receptors and regulatory potentials on target genes expression in Vas_ECs at e14.5 (analysis of *Wt1* mutant). (I) The predicted activity of epicardial cell derived ligands and their regulatory potentials on target genes expression in Vas_ECs at e17.5, and (J) the ligands' expression pattern in different cell types and (K) their prior interaction potentials with receptors (analysis of *Tbx18* mutant).

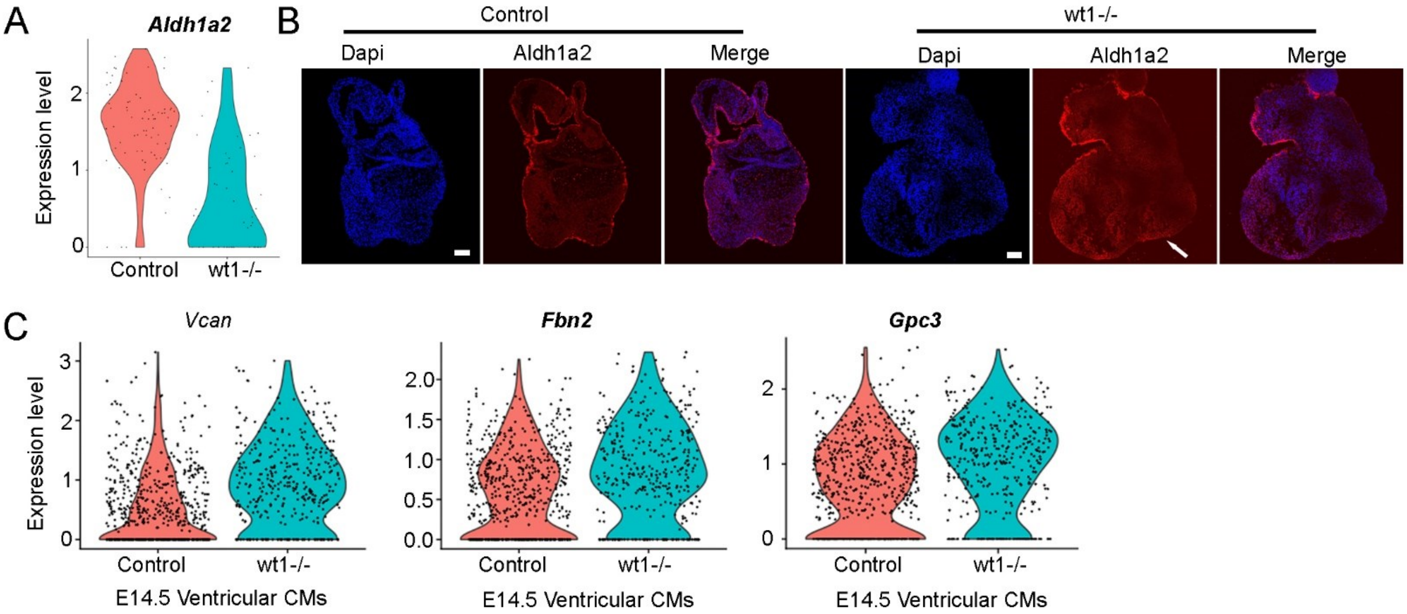

Fig S32. Gene expression analysis of the Wt1 mutant hearts. (A) ScRNA-seq data revealed a reduction of *Aldh1a2* expression in Wt1 mutant epicardial cells. (B) Antibody staining confirmed the reduction of *Aldh1a2* in *Wt1* mutant ventricular cells (arrow). However, the reduction was not observed in atrial epicardial cells. The staining experiments were repeated on three heart sections with similar results. (C) ScRNA-seq data revealed an upregulation of Tgfb3 target genes *Vcan*, *Fbn2*, and *Gpc3* in *Wt1* mutant ventricular CMs compared to control CMs. Scale bar=100 µm.

Table S1. The sequence of barcodes used in the MULTI-seq experiments.

Anchor LMO	5’-TGGAATTCTCGGGTGCCAAGGgtaacgatccagctgtcact-Lipid-3’
Co-Anchor LMO	5’-Lipid-AGTGACAGCTGGATCGTTAC-3’
MULTI-seq Additive Primer	5’-CTTGGCACCCGAGAATTCC-3’
TruSeq RPIX Primer	5’-CAAGCAGAAGACGGCATACGAGATNNNNNNGTGACTGGAGTT CCTTGGCACCCGAGAATTCCA-3’
TruSeq Universal Adapter Primer	5’-AATGATACGGCGACCACCGAGATCTACACTCTTTCCCTACACGA CGCTCTTCCGATCT-3’
BC1	CCTTGGCACCCGAGAATTCCAGGAGAAGAAAAAAAAAAAAAAAAA AAAAAAAAAAAAAA
BC2	CCTTGGCACCCGAGAATTCCACCACAATGAAAAAAAAAAAAAAAAA AAAAAAAAAAAAA
BC3	CCTTGGCACCCGAGAATTCCATGAGACCTAAAAAAAAAAAAAAAAA AAAAAAAAAAAAA
BC4	CCTTGGCACCCGAGAATTCCAGCACACGCAAAAAAAAAAAAAAAA AAAAAAAAAAAAAA
BC5	CCTTGGCACCCGAGAATTCCAAGAGAGAGAAAAAAAAAAAAAAAA AAAAAAAAAAAAAA
BC6	CCTTGGCACCCGAGAATTCCATCACAGCAAAAAAAAAAAAAAAAA AAAAAAAAAAAAA
BC7	CCTTGGCACCCGAGAATTCCAGAAAAGGGAAAAAAAAAAAAAAAA AAAAAAAAAAAAAA
BC8	CCTTGGCACCCGAGAATTCCACGAGATTCAAAAAAAAAAAAAAAAA AAAAAAAAAAAAA
BC9	CCTTGGCACCCGAGAATTCCAGTAGCACTAAAAAAAAAAAAAAAAA AAAAAAAAAAAAA
BC10	CCTTGGCACCCGAGAATTCCACGACCAGCAAAAAAAAAAAAAAAA AAAAAAAAAAAAAA
BC11	CCTTGGCACCCGAGAATTCCATTAGCCAGAAAAAAAAAAAAAAAAA AAAAAAAAAAAAA
BC12	CCTTGGCACCCGAGAATTCCAGGACCCCAAAAAAAAAAAAAAAAA AAAAAAAAAAAAAA
BC13	CCTTGGCACCCGAGAATTCCACCAACCGGAAAAAAAAAAAAAAAA AAAAAAAAAAAAAA
BC14	CCTTGGCACCCGAGAATTCCATGACCGATAAAAAAAAAAAAAAAAA AAAAAAAAAAAAA
BC15	CCTTGGCACCCGAGAATTCCAGCAACGCCAAAAAAAAAAAAAAAA AAAAAAAAAAAAAA
BC16	CCTTGGCACCCGAGAATTCCACAATCGGTAAAAAAAAAAAAAAAAA AAAAAAAAAAAAA
BC17	CCTTGGCACCCGAGAATTCCAATAGCGTCAAAAAAAAAAAAAAAAA AAAAAAAAAAAAA
BC18	CCTTGGCACCCGAGAATTCCAGAATCTCGAAAAAAAAAAAAAAAAA AAAAAAAAAAAAA
BC19	CCTTGGCACCCGAGAATTCCACTAGCTGAAAAAAAAAAAAAAAAAA AAAAAAAAAAAAA
BC20	CCTTGGCACCCGAGAATTCCAAGACCTTGAAAAAAAAAAAAAAAAA AAAAAAAAAAAAA
BC21	CCTTGGCACCCGAGAATTCCAGAAGGAAGAAAAAAAAAAAAAAAA AAAAAAAAAAAAAA

BC22	CCTTGGCACCCGAGAATTCCACTACGACAAAAAAAAAAAAAAAAAA<br>AAAAAAAAAAAAA
BC23	CCTTGGCACCCGAGAATTCCAAGAAGAGGAAAAAAAAAAAAAAAA<br>AAAAAAAAAAAAAA
BC24	CCTTGGCACCCGAGAATTCCAGTACGCATAAAAAAAAAAAAAAAAA<br>AAAAAAAAAAAAA
BC25	CCTTGGCACCCGAGAATTCCACGAAGCCCAAAAAAAAAAAAAAAA<br>AAAAAAAAAAAAAA
BC26	CCTTGGCACCCGAGAATTCCAACATGCGTAAAAAAAAAAAAAAAAA<br>AAAAAAAAAAAAA
BC27	CCTTGGCACCCGAGAATTCCATAAGGCTCAAAAAAAAAAAAAAAAA<br>AAAAAAAAAAAAA
BC28	CCTTGGCACCCGAGAATTCCAGGAAGGAAAAAAAAAAAAAAAAAA<br>AAAAAAAAAAAAAA
BC29	CCTTGGCACCCGAGAATTCCACCATGGCGAAAAAAAAAAAAAAAA<br>AAAAAAAAAAAAAA
BC30	CCTTGGCACCCGAGAATTCCAAAAGGGGAAAAAAAAAAAAAAAAA<br>AAAAAAAAAAAAAA
BC31	CCTTGGCACCCGAGAATTCCATTACGGTGAAAAAAAAAAAAAAAAA<br>AAAAAAAAAAAAA
BC32	CCTTGGCACCCGAGAATTCCAGCATGTACAAAAAAAAAAAAAAAAA<br>AAAAAAAAAAAAA
BC33	CCTTGGCACCCGAGAATTCCACAAGGTCTAAAAAAAAAAAAAAAAA<br>AAAAAAAAAAAAA
BC34	CCTTGGCACCCGAGAATTCCAATACGTGCAAAAAAAAAAAAAAAAA<br>AAAAAAAAAAAAA
BC35	CCTTGGCACCCGAGAATTCCAGCAGTATCAAAAAAAAAAAAAAAAA<br>AAAAAAAAAAAAA
BC36	CCTTGGCACCCGAGAATTCCAAGATTCCGAAAAAAAAAAAAAAAAA<br>AAAAAAAAAAAAA
BC37	CCTTGGCACCCGAGAATTCCATCAGTCGAAAAAAAAAAAAAAAAAA<br>AAAAAAAAAAAAA
BC38	CCTTGGCACCCGAGAATTCCAGAACTCTGAAAAAAAAAAAAAAAAA<br>AAAAAAAAAAAAA
BC39	CCTTGGCACCCGAGAATTCCACGATTGACAAAAAAAAAAAAAAAAA<br>AAAAAAAAAAAAA
BC40	CCTTGGCACCCGAGAATTCCAACAGTGCTAAAAAAAAAAAAAAAAA<br>AAAAAAAAAAAAA
BC41	CCTTGGCACCCGAGAATTCCATAACTGGCAAAAAAAAAAAAAAAAA<br>AAAAAAAAAAAAA
BC42	CCTTGGCACCCGAGAATTCCACCAGTTAGAAAAAAAAAAAAAAAAA<br>AAAAAAAAAAAAA
BC43	CCTTGGCACCCGAGAATTCCAGGCTAACTAAAAAAAAAAAAAAAAA<br>AAAAAAAAAAAAA
BC44	CCTTGGCACCCGAGAATTCCACCCGAAGCAAAAAAAAAAAAAAAA<br>AAAAAAAAAAAAAA
BC45	CCTTGGCACCCGAGAATTCCATGCTACAGAAAAAAAAAAAAAAAAA<br>AAAAAAAAAAAAA
BC46	CCTTGGCACCCGAGAATTCCAGCCGACCAAAAAAAAAAAAAAAAA<br>AAAAAAAAAAAAAA
BC47	CCTTGGCACCCGAGAATTCCACACCACGGAAAAAAAAAAAAAAAA<br>AAAAAAAAAAAAAA

BC48	CCTTGGCACCCGAGAATTCCATCCGAGATAAAAAAAAAAAAAAAAAAAAAAAAAAAAAA
BC49	CCTTGGCACCCGAGAATTCCAGACCAGCCAAAAAAAAAAAAAAAAAAAAAAAAAAAAAA
BC50	CCTTGGCACCCGAGAATTCCACTCAAGGTAAAAAAAAAAAAAAAAAAAAAAAAAAAAAA
BC51	CCTTGGCACCCGAGAATTCCAAGCTAGTCAAAAAAAAAAAAAAAAAAAAAAAAAAAAAA
BC52	CCTTGGCACCCGAGAATTCCAGTCAATCGAAAAAAAAAAAAAAAAAAAAAAAAAAAAAA
BC53	CCTTGGCACCCGAGAATTCCACGCTATGAAAAAAAAAAAAAAAAAAAAAAAAAAAAAAA
BC54	CCTTGGCACCCGAGAATTCCAACCGATTGAAAAAAAAAAAAAAAAAAAAAAAAAAAAAA
BC55	CCTTGGCACCCGAGAATTCCAGTCTCAAGAAAAAAAAAAAAAAAAAAAAAAAAAAAAAA
BC56	CCTTGGCACCCGAGAATTCCACGCGCACAAAAAAAAAAAAAAAAAAAAAAAAAAAAAA
BC57	CCTTGGCACCCGAGAATTCCAACCCCAGGAAAAAAAAAAAAAAAAAAAAAAAAAAAAAA
BC58	CCTTGGCACCCGAGAATTCCAGGCGCCATAAAAAAAAAAAAAAAAAAAAAAAAAAAAAA
BC59	CCTTGGCACCCGAGAATTCCAAACACCGTAAAAAAAAAAAAAAAAAAAAAAAAAAAAAA
BC60	CCTTGGCACCCGAGAATTCCATTCTCCTCAAAAAAAAAAAAAAAAAAAAAAAAAAAAAA
BC61	CCTTGGCACCCGAGAATTCCAGCCCCGAAAAAAAAAAAAAAAAAAAAAAAAAAAAAAA
BC62	CCTTGGCACCCGAGAATTCCACACACGCGAAAAAAAAAAAAAAAAAAAAAAAAAAAAAA
BC63	CCTTGGCACCCGAGAATTCCAATCTCGGAAAAAAAAAAAAAAAAAAAAAAAAAAAAAAA
BC64	CCTTGGCACCCGAGAATTCCATGCGCGTGAAAAAAAAAAAAAAAAAAAAAAAAAAAAAA
BC65	CCTTGGCACCCGAGAATTCCAGACACTACAAAAAAAAAAAAAAAAAAAAAAAAAAAAAA
BC66	CCTTGGCACCCGAGAATTCCACTCTCTCTAAAAAAAAAAAAAAAAAAAAAAAAAAAAAA
BC67	CCTTGGCACCCGAGAATTCCAAGCGCTGCAAAAAAAAAAAAAAAAAAAAAAAAAAAAAA
BC68	CCTTGGCACCCGAGAATTCCATCCCCTTTAAAAAAAAAAAAAAAAAAAAAAAAAAAAAA
BC69	CCTTGGCACCCGAGAATTCCACTCGGAATAAAAAAAAAAAAAAAAAAAAAAAAAAAAAA
BC70	CCTTGGCACCCGAGAATTCCAAGCCGACCAAAAAAAAAAAAAAAAAAAAAAAAAAAAAA
BC71	CCTTGGCACCCGAGAATTCCATCCAGAGTAAAAAAAAAAAAAAAAAAAAAAAAAAAAAA
BC72	CCTTGGCACCCGAGAATTCCAGACTGATCAAAAAAAAAAAAAAAAAAAAAAAAAAAAAA

Table S2. The sequence of H probes that were used for single molecular in situ hybridization (PLISH) analysis.

**Name**	**Sequence**
cy5-mApela-Right-1	atgtgtggaaagacggccatTTATACGTCGAGTTGAACGTCGTAACA
cy5-mApela-left-1	TAGCGCTAACAACTTACGTCGTTATGaggacgtgatgtactggtat
cy5-mApela-Right-2	attcagacaaacgcatgtgcTTATACGTCGAGTTGAACGTCGTAACA
cy5-mApela-left-2	TAGCGCTAACAACTTACGTCGTTATGtttaacctcgtctgttttcc
cy5-mApela-Right-3	tgaaaagccatccacggtacTTATACGTCGAGTTGAACGTCGTAACA
cy5-mApela-left-3	TAGCGCTAACAACTTACGTCGTTATGtctgaaacagcctctgttgc
cy5-mApela-Right-4	cgtctgtaaatcgcagtatcTTATACGTCGAGTTGAACGTCGTAACA
cy5-mApela-left-4	TAGCGCTAACAACTTACGTCGTTATGtgtccggctcaccccacatc
cy5-mApela-Right-5	ccattcaggcacgcagtaacTTATACGTCGAGTTGAACGTCGTAACA
cy5-mApela-left-5	TAGCGCTAACAACTTACGTCGTTATGttaagcgggaagtctctgca
cy5-mLrrn4-Right-1	gttaccagaaggccagttttTTATACGTCGAGTTGAACGTCGTAACA
cy5-mLrrn4-left-1	TAGCGCTAACAACTTACGTCGTTATGaactgtaactctggaaatac
cy5-mLrrn4-Right-2	tactccgtatatccctcagtTTATACGTCGAGTTGAACGTCGTAACA
cy5-mLrrn4-left-2	TAGCGCTAACAACTTACGTCGTTATGaccaagtgagatactacact
cy5-mLrrn4-Right-3	tactgagggatatacggagtTTATACGTCGAGTTGAACGTCGTAACA
cy5-mLrrn4-left-3	TAGCGCTAACAACTTACGTCGTTATGactgaccaatgtaaaggatg
cy5-mLrrn4-Right-4	ggagctagtaagacaggcgtTTATACGTCGAGTTGAACGTCGTAACA
cy5-mLrrn4-left-4	TAGCGCTAACAACTTACGTCGTTATGagtgagaacaactataggag
cy5-mLrrn4-Right-5	cagctcaaggccaacagaacTTATACGTCGAGTTGAACGTCGTAACA
cy5-mLrrn4-left-5	TAGCGCTAACAACTTACGTCGTTATGtcattgtgctaaaatcgggc
cy5-mAdamts8-Right-1	AGGTCAGCACATAAGAGGGCTTATACGTCGAGTTGAACGTCGTAACA
cy5-mAdamts8-left-1	TAGCGCTAACAACTTACGTCGTTATGTGATAGTGCAGCTTGCTGAG
cy5-mAdamts8-Right-2	AGTGGGTAATAGGTAGGGACTTATACGTCGAGTTGAACGTCGTAACA
cy5-mAdamts8-left-2	TAGCGCTAACAACTTACGTCGTTATGTGTTCCAGGTTAATAGCACC
cy5-mAdamts8-Right-3	CTAGACAATTTCATTGGTGTTTATACGTCGAGTTGAACGTCGTAACA
cy5-mAdamts8-left-3	TAGCGCTAACAACTTACGTCGTTATGAAGATCACACTTTAGTTCGG
cy5-mAdamts8-Right-4	ATTTTTCCCCGCTGTGAGGTTTATACGTCGAGTTGAACGTCGTAACA
cy5-mAdamts8-left-4	TAGCGCTAACAACTTACGTCGTTATGAGATTGATTATTGGGGAAAC
cy5-mAdamts8-Right-5	CGGACGCAGATGGACAGAGTTTATACGTCGAGTTGAACGTCGTAACA
cy5-mAdamts8-left-5	TAGCGCTAACAACTTACGTCGTTATGATCCGGTCCACACAGAGTGC
cy5-mDgit4l-Right-1	ccagagaactgctaacgggtTTATACGTCGAGTTGAACGTCGTAACA
cy5-mDgit4l-left-1	TAGCGCTAACAACTTACGTCGTTATGaaaagatctatgtccatgcc
cy5-mDgit4l-Right-2	ggcccagtcttgtgaagactTTATACGTCGAGTTGAACGTCGTAACA
cy5-mDgit4l-left-2	TAGCGCTAACAACTTACGTCGTTATGagctccgtggtacctcccca
cy5-mDgit4l-Right-3	ttgctgaatagaacagagttTTATACGTCGAGTTGAACGTCGTAACA
cy5mDgit4l-left-3	TAGCGCTAACAACTTACGTCGTTATGacgtggaaggtaaattgtct
cy5-mDgit4l-Right-4	tttcatatctagtctgtggtTTATACGTCGAGTTGAACGTCGTAACA
cy5-mDgit4l-left-4	TAGCGCTAACAACTTACGTCGTTATGagcaacaacatctcagagtc
cy5-mDgit4l-Right-5	acaaagcagcaatgtgacctTTATACGTCGAGTTGAACGTCGTAACA
cy5-mDgit4l-left-5	TAGCGCTAACAACTTACGTCGTTATGagtggatccgaaagggacgg
cy5-mAdm-Right-1	tagttccctcttcccacgacTTATACGTCGAGTTGAACGTCGTAACA
cy5-mAdm-left-1	TAGCGCTAACAACTTACGTCGTTATGttagcgcccacttattccac

cy5-mAdm-Right-2	tctgggtaggaactgtcgtcTTATACGTCGAGTTGAACGTCGTAACA
cy5-mAdm-left-2	TAGCGCTAACAACTTACGTCGTTATGtcatcagcgagtcccgtagg
cy5-mAdm-Right-3	gcttcgctctgattgctggcTTATACGTCGAGTTGAACGTCGTAACA
cy5-mAdm-left-3	TAGCGCTAACAACTTACGTCGTTATGttgtagggggccagttgtgt
cy5-mAdm--Right-4	tcaatgctgtcacccgcaccTTATACGTCGAGTTGAACGTCGTAACA
cy5-mAdm--left-4	TAGCGCTAACAACTTACGTCGTTATGtatatcctaaagagtctgga
cy5-mAdm--Right-5	cgcaggcgccaacgggatacTTATACGTCGAGTTGAACGTCGTAACA
cy5-mAdm--left-5	TAGCGCTAACAACTTACGTCGTTATGtcgcccgactgttcaatgct
cy5-mBmp10-Right-1	cttcttccaggggcgactgcTTATACGTCGAGTTGAACGTCGTAACA
cy5-mBmp10-left-1	TAGCGCTAACAACTTACGTCGTTATGtcaaggcccataatggggct
cy5-mBmp10-Right-2	tgaagtcaataccatcttgcTTATACGTCGAGTTGAACGTCGTAACA
cy5-mBmp10-left-2	TAGCGCTAACAACTTACGTCGTTATGtccgtgaagatatcatcaaa
cy5-mBmp10-Right-3	cctcgctaccgtctgcactcTTATACGTCGAGTTGAACGTCGTAACA
cy5-mBmp10-left-3	TAGCGCTAACAACTTACGTCGTTATGtctagtacctcaaaaatggt
cy5-mBmp10-Right-4	ctcctcgctaccgtctgcacTTATACGTCGAGTTGAACGTCGTAACA
cy5-mBmp10-left-4	TAGCGCTAACAACTTACGTCGTTATGtctctagtacctcaaaaatg
cy5-mBmp10-Right-5	ctgttggttccgtagatctcTTATACGTCGAGTTGAACGTCGTAACA
cy5-mBmp10-left-5	TAGCGCTAACAACTTACGTCGTTATGtgttgatactaagaccagca
cy5-mBmp10-Right-6	cactgttggttccgtagatcTTATACGTCGAGTTGAACGTCGTAACA
cy5-mBmp10-left-6	TAGCGCTAACAACTTACGTCGTTATGtctgttgatactaagaccag
cy5-mMest-Right-1	aagaaatcaagggcgatcacTTATACGTCGAGTTGAACGTCGTAACA
cy5-mMest-left-1	TAGCGCTAACAACTTACGTCGTTATGtcgatggaacctcagggtca
cy5-mMest-Right-2	ccgaccacaccgacagaatcTTATACGTCGAGTTGAACGTCGTAACA
cy5-mMest-left-2	TAGCGCTAACAACTTACGTCGTTATGttggtagaagatgcgtaggc
cy5-mMest-Right-3	gccgaggcgcccgcagcgatTTATACGTCGAGTTGAACGTCGTAACA
cy5-mMest-left-3	TAGCGCTAACAACTTACGTCGTTATGacaggatcggaggtggcgtc
cy5-mMest-Right-4	attaatgtactgtaaaagacTTATACGTCGAGTTGAACGTCGTAACA
cy5-mMest-left-4	TAGCGCTAACAACTTACGTCGTTATGtgtcgatgaccaggttgccg
cy5-mMest-Right-5	ctgcatttgggctatggaacTTATACGTCGAGTTGAACGTCGTAACA
cy5-mMest-left-5	TAGCGCTAACAACTTACGTCGTTATGtgcgcaagcgatctcggcgc
cy5-mSfrp2-Right-1	attcgcctgacatggcaaccTTATACGTCGAGTTGAACGTCGTAACA
cy5-mSfrp2-left-1	TAGCGCTAACAACTTACGTCGTTATGtaagtgcacggtataagcca
cy5-mSfrp2-Right-2	ggccggcaggaggtggtcgc TTATACGTCGAGTTGAACGTCGTAACA
cy5-mSfrp2-left-2	TAGCGCTAACAACTTACGTCGTTATGtactagcgagggggatgcag
cy5-mSfrp2-Right-3	cacggctggatggtctcatcTTATACGTCGAGTTGAACGTCGTAACA
cy5-mSfrp2-left-3	TAGCGCTAACAACTTACGTCGTTATGtaggtcgtcgagacagacag
cy5-mSfrp2-Right-4	acgcatgcaatggtcagagcTTATACGTCGAGTTGAACGTCGTAACA
cy5-mSfrp2-left-4	TAGCGCTAACAACTTACGTCGTTATGtcatgagggcataaaacgag
cy5-mSfrp2-Right-5	gcagccgcatgttctggtacTTATACGTCGAGTTGAACGTCGTAACA
cy5-mSfrp2-left-5	TAGCGCTAACAACTTACGTCGTTATGtcgatgccgtggcacagctg
cy5-mCldn5-Right-1	cctacttcaccgatgaaatcTTATACGTCGAGTTGAACGTCGTAACA
cy5-mCldn5-left-1	TAGCGCTAACAACTTACGTCGTTATGtgagcgttccacccacgtcg
cy5-mCldn5-Right-2	agcgctcctcccgtgagtgcTTATACGTCGAGTTGAACGTCGTAACA
cy5-mCldn5-left-2	TAGCGCTAACAACTTACGTCGTTATGtacccgtgccttaactgggc
cy5-mCldn5-Right-3	ctggacattaaggcagcatcTTATACGTCGAGTTGAACGTCGTAACA
cy5-mCldn5-left-3	TAGCGCTAACAACTTACGTCGTTATGtagtgcccccaggatctcag

cy5-mCldn5-Right-4	cggtctttcccatcgcgggcTTATACGTCGAGTTGAACGTCGTAACA
cy5-mCldn5-left-4	TAGCGCTAACAACTTACGTCGTTATGttagctgcgggaagagcccc
cy5-mCldn5-Right-5	gcaccgtcggatcatagaacTTATACGTCGAGTTGAACGTCGTAACA
cy5-mCldn5-left-5	TAGCGCTAACAACTTACGTCGTTATGtcgcggacaacgatgttggc
cy5-mCol23a1-Right-1	ctgtgacaactcaaagctttTTATACGTCGAGTTGAACGTCGTAACA
cy5-mCol23a1-left-1	TAGCGCTAACAACTTACGTCGTTATGaaggacagggaatctagtaa
cy5-mCol23a1-Right-2	cagctagtaaccatcggaatTTATACGTCGAGTTGAACGTCGTAACA
cy5-mCol23a1-left-2	TAGCGCTAACAACTTACGTCGTTATGaaaactcacccagaggtgac
cy5-mCol23a1-Right-3	acagatgagtggtgttatttTTATACGTCGAGTTGAACGTCGTAACA
cy5-mCol23a1-left-3	TAGCGCTAACAACTTACGTCGTTATGaagatgccgtaacaagggct
cy5-mCol23a1-Right-4	gccctaacgatgttgttcttTTATACGTCGAGTTGAACGTCGTAACA
cy5-mCol23a1-left-4	TAGCGCTAACAACTTACGTCGTTATGaggatgaaggaggagtgctt
cy5-mCol23a1-Right-5	ttcaggttcatggtgtccgtTTATACGTCGAGTTGAACGTCGTAACA
cy5-mCol23a1-left-5	TAGCGCTAACAACTTACGTCGTTATGaccatttactaaggaggcct
cy5-mFoxc1-Right-1	agttgttcaagccgatccgcTTATACGTCGAGTTGAACGTCGTAACA
cy5-mFoxc1-left-1	TAGCGCTAACAACTTACGTCGTTATGtgagactcgaacatttcccg
cy5-mFoxc1-Right-2	ggaggaacgttcggatcaacTTATACGTCGAGTTGAACGTCGTAACA
cy5-mFoxc1-left-2	TAGCGCTAACAACTTACGTCGTTATGttccaggcgcagtcggggca
cy5-mFoxc1-Right-3	cagtctcttgcgccggccgcTTATACGTCGAGTTGAACGTCGTAACA
cy5-mFoxc1-left-3	TAGCGCTAACAACTTACGTCGTTATGttaaggaagcattgggaaaa
cy5-mFoxc1-Right-4	gggctgcggctgcggcgtatTTATACGTCGAGTTGAACGTCGTAACA
cy5-mFoxc1-left-4	TAGCGCTAACAACTTACGTCGTTATGaaggcccgtaggcgcgcgcc
cy5-mFoxc1-Right-5	gttccattccatttggctctTTATACGTCGAGTTGAACGTCGTAACA
cy5-mFoxc1-left-5	TAGCGCTAACAACTTACGTCGTTATGaagagtgccgggaatagggt
cy5-mPlvap-Right-1	ggaagctggcgttgatgcgcTTATACGTCGAGTTGAACGTCGTAACA
cy5-mPlvap-left-1	TAGCGCTAACAACTTACGTCGTTATGtccatctcacgtcgcgtagt
cy5-mPlvap-Right-2	ctaagatccacccggaacgcTTATACGTCGAGTTGAACGTCGTAACA
cy5-mPlvap-left-2	TAGCGCTAACAACTTACGTCGTTATGtcatctacaattgaggccct
cy5-mPlvap-Right-3	cgcgctctagctccaacttc TTATACGTCGAGTTGAACGTCGTAACA
cy5-mPlvap-left-3	TAGCGCTAACAACTTACGTCGTTATGtggcgccgcagttctgcatt
cy5-mPlvap-Right-4	atctggaaaccctagtggccTTATACGTCGAGTTGAACGTCGTAACA
cy5-mPlvap-left-4	TAGCGCTAACAACTTACGTCGTTATGtttaagagggaggctggaaa
cy5-mPlvap-Right-5	cggcatcagctggtagtggtTTATACGTCGAGTTGAACGTCGTAACA
cy5-mPlvap-left-5	TAGCGCTAACAACTTACGTCGTTATGagggcagggtttccaaggta
cy3-mWt1-Right-1	cggaacccatgaggtgcggcTTATACGTCGAGTTGACCGACGTATTG
cy3-mWt1-left-1	TATTCGTTCGAACTTACGTCGTTATGtcggatgcggacggttctcc
cy3-mWt1-Right-2	aaagtgaccgtgctgtatccTTATACGTCGAGTTGACCGACGTATTG
cy3-mWt1-left-2	TATTCGTTCGAACTTACGTCGTTATGttggttgcggatggtaggct
cy3-mWt1-Right-3	gttactcaaaacctatgagcTTATACGTCGAGTTGACCGACGTATTG
cy3-mWt1-left-3	TATTCGTTCGAACTTACGTCGTTATGtttaacaatcgcctgtagaa
cy3-mWt1-Right-4	cacacagtgatgcagctaatTTATACGTCGAGTTGACCGACGTATTG
cy3-mWt1-left-4	TATTCGTTCGAACTTACGTCGTTATGaccatacacctcctaatttt
cy3-mNpr3-Right-1	ttgcaaggagagctgttggtTTATACGTCGAGTTGACCGACGTATTG
cy3-mNpr3-left-1	TATTCGTTCGAACTTACGTCGTTATGatgctccacgattctggtct
cy3-mNpr3-Right-2	taagctgaacagacttctgtTTATACGTCGAGTTGACCGACGTATTG
cy3-mNpr3-left-2	TATTCGTTCGAACTTACGTCGTTATGacatcttcagccagactagc

cy3-mNpr3-Right-3	cctagctcactgtccaagctTTATACGTCGAGTTGACCGACGTATTG
cy3-mNpr3-left-3	TATTCGTTCGAACTTACGTCGTTATGatgcatcatttgtggtggct
cy3-mNpr3-Right-4	tagaataggaagctatatgtTTATACGTCGAGTTGACCGACGTATTG
cy3-mNpr3-left-4	TATTCGTTCGAACTTACGTCGTTATGagctggtgcagctttgactc
cy3-mFabp4-Right-1	ctgcagcacaggagggtgctTTATACGTCGAGTTGACCGACGTATTG
cy3-mFabp4-left-1	TATTCGTTCGAACTTACGTCGTTATGatgagcctctgaagtccaga
cy3-mFabp4-Right-2	gtggcaaagcccactcccacTTATACGTCGAGTTGACCGACGTATTG
cy3-mFabp4-left-2	TATTCGTTCGAACTTACGTCGTTATGttctttcatgtaatcatcga
cy3-mFabp4-Right-3	tggtgaccaaatccccatttTTATACGTCGAGTTGACCGACGTATTG
cy3-mFabp4-left-3	TATTCGTTCGAACTTACGTCGTTATGacgctgatgatcatgttggg
cy3-mFabp4-Right-4	gtggaagtcacgcctttcatTTATACGTCGAGTTGACCGACGTATTG
cy3-mFabp4-left-4	TATTCGTTCGAACTTACGTCGTTATGaacacattccaccaccagct

Table S3. The sequence of qPCR primers and mouse genotyping primers.

Atf3 Forward Primer	GAGGATTTTGCTAACCTGACACC
Atf3 Reverse Primer	TTGACGGTAACTGACTCCAGC
Eno3 Forward Primer	CACAGCCAAGGGTCGATTCC
Eno3 Reverse Primer	CCCAGGTATCGTGCTTTGTCT
Klf9 Forward Primer	TTATTGCACGCTGGTCACTATC
Klf9 Reverse Primer	CTCATCGGGACTCTCCAGAC
Fhl2 Forward Primer	ATGACTGAACGCTTTGACTGC
Fhl2 Reverse Primer	CGATGGGTGTTCCACACTCC
Rps2 Forward Primer	GGGGCTCGTGGAGGTAAAG
Rps2 Reverse Primer	TCTCAGACTCCTTAATGGGCAG
Ranbp1 Forward Primer	CGAGGACCATGATACTTCCACA
Ranbp1 Reverse Primer	CCTCCAGCGTTTTAATTTCTTGC
Per1 Forward Primer	GAATTGGAGCATATCACATCCGA
Per1 Reverse Primer	CCCGAAACACATCCCGTTTG
Gapdh Forward Primer	GGATTTGGTCGTATTGGG
Gapdh Reverse Primer	GGAAGATGGTGATGGGATT
GM8797 Forward Primer	GGCAAGACCATCACCCTAGA
GM8797 Reverse Primer	TAATAGCCACCCCTCAGACG
GM10260 Forward Primer	GATCCCAGACTGGTTCCTGA
GM10260 Reverse Primer	TAAGAGCAAAGGCCCAGAGA
Fbn2 Forward Primer	CAACTCCGAAGGAAGCTACG
Fbn2 Reverse Primer	AAGAGCCCTTCGTGTTCTCA
Gpc3 Forward Primer	CCAACATGCTGCTCAAGAAA
Gpc3 Reverse Primer	CTGGAAAGAGGCTGTCGAAC
Vcan Forward Primer	GGCCAACAGTGGTTTCAAGT
Vcan Reverse Primer	TTCTGTGAAGGCTGTCGATG

Mouse genotyping primers

Tbx18-Cre wt Forward Primer	AACGCCAGAGAAAGAGGAAAC
Tbx18-Cre wt Reverse Primer	TCAGTGCCTCCACAGAGAAG
Tbx18-Cre mutant Forward Primer	AACGCCAGAGAAAGAGGAAAC
Tbx18-Cre mutant Reverse Primer	AGGCAAATTTTGGTGTACGG
Wt1-Cre wt Forward Primer	CCTACCATCCGCAACCAAG

Wt1-Cre wt Reverse Primer	CCCTGTCCGCTACTTTCAGA
Wt1-Cre mutant Forward Primer	ATCGCAGGAGCGGAGAAC
Wt1-Cre mutant Reverse Primer	GAACTTCAGGGTCAGCTTGC
