# Supplementary material for: Single-cell transcriptomic analysis identifies murine heart molecular features at embryonic and neonatal stages
Source: Nat Commun. 2022 Dec 27;13:7960. doi: 10.1038/s41467-022-35691-7 (PMC9794824; doi:10.1038/s41467-022-35691-7)
Supplement: Supplementary file 3 — Description of Additional Supplementary Files [file 41467_2022_35691_MOESM3_ESM.pdf]

Description of Additional Supplementary Files

File Name: Supplementary Data 1
Description: The genes differentially expressed between mouse strains, which were identified through comparative analysis of cell type, stage, zone matched, and G1 phased cells.

File Name: Supplementary Data 2
Description: The genes and their enriched pathways in each gene module.

File Name: Supplementary Data 3
Description: The stage-specific transcription factors in each cell type.

File Name: Supplementary Data 4
Description: The predicted regulatory transcription factors for the genes in each module based on motif enrichment analysis.

File Name: Supplementary Data 5
Description: The genes that are differentially expressed in LV and RV CMs. The analysis was carried out in Seurat using a default non-parameteric Wilcoxon rank sum test. The p-value was presented without adjustment.

File Name: Supplementary Data 6
Description: The genes that are differentially expressed in each cell type. The analysis was carried out in Seurat using a default non-parametric Wilcoxon rank sum test. The p-value was presented without adjustment.

File Name: Supplementary Data 7
Description: The group of curated known CHD genes

File Name: Supplementary Data 8
Description: The complete list of ligand-receptor interactions and their expression stages in CD1 single cells.

File Name: Supplementary Data 9
Description: The transcription factors predicted to regulate the target genes in Fig 7C.

File Name: Supplementary Data 10
Description: The ligand-receptor interactions between epicardial cells and the other cell types at different stages. The p-values were calculated in CellPhoneDB using a statistical framework they developed.

File Name: Supplementary Data 11
Description: The genes that are abnormally expressed in Wt1 mutants at each cell type and stage. The analysis was carried out in Seurat using a default non-parametric Wilcoxon rank sum test. The p-value was presented without adjustment.

File Name: Supplementary Data 12
Description: The genes that are abnormally expressed in Tbx18 mutants at each cell type and stage. The analysis was carried out in Seurat using a default non-parametric Wilcoxon rank sum test. The p-value was presented without adjustment.

File Name: Supplementary Data 13
Description: The genes that are abnormally expressed in both Wt1 and Tbx18 mutant epicardial cells.

File Name: Supplementary Data 14
Description: The genes that are abnormally expressed at both E14.5 and E17.5 in Tbx18 mutant epicardial cells.

File Name: Supplementary Software 1
Description: The computational codes used to generate the results of the study.
