# Supplementary material for: Single-cell transcriptomic analysis identifies murine heart molecular features at embryonic and neonatal stages
Source: Nat Commun. 2022 Dec 27;13:7960. doi: 10.1038/s41467-022-35691-7 (PMC9794824; doi:10.1038/s41467-022-35691-7)
Supplement: Supplementary file 19 — Reporting Summary [file 41467_2022_35691_MOESM19_ESM.pdf]

nature portfolio

Corresponding author(s): Guang Li
Last updated by author(s): Dec 10, 2022

Reporting Summary

Nature Portfolio wishes to improve the reproducibility of the work that we publish. This form provides structure for consistency and transparency in reporting. For further information on Nature Portfolio policies, see our Editorial Policies and the Editorial Policy Checklist.

Statistics

For all statistical analyses, confirm that the following items are present in the figure legend, table legend, main text, or Methods section.

n/a	Confirmed	
☐	☒	The exact sample size (*n*) for each experimental group/condition, given as a discrete number and unit of measurement
☒	☐	A statement on whether measurements were taken from distinct samples or whether the same sample was measured repeatedly
☐	☒	The statistical test(s) used AND whether they are one- or two-sided<br>*Only common tests should be described solely by name; describe more complex techniques in the Methods section.*
☐	☒	A description of all covariates tested
☒	☐	A description of any assumptions or corrections, such as tests of normality and adjustment for multiple comparisons
☐	☒	A full description of the statistical parameters including central tendency (e.g. means) or other basic estimates (e.g. regression coefficient) AND variation (e.g. standard deviation) or associated estimates of uncertainty (e.g. confidence intervals)
☐	☒	For null hypothesis testing, the test statistic (e.g. *F*, *t*, *r*) with confidence intervals, effect sizes, degrees of freedom and *P* value noted<br>*Give P values as exact values whenever suitable.*
☒	☐	For Bayesian analysis, information on the choice of priors and Markov chain Monte Carlo settings
☐	☒	For hierarchical and complex designs, identification of the appropriate level for tests and full reporting of outcomes
☐	☒	Estimates of effect sizes (e.g. Cohen's *d*, Pearson's *r*), indicating how they were calculated

Our web collection on statistics for biologists contains articles on many of the points above.

Software and code

Policy information about availability of computer code

Data collection	10X Genomics Chromium 120223; Chromium single cell 3' GEM, Library & Gel Bead Kit V3 PN-1000092; Illumina HiSeq X;
Data analysis	Cell Ranger Version 3.1; Imaris 9.9.0; R packages: deMULTIplex 1.0.2, Seurat v4.0, Monocle v3, Nichnet 1.1.0; Python package: CellPhoneDB v3, cellchat 1.5.0, RcisTarget 1.16.

For manuscripts utilizing custom algorithms or software that are central to the research but not yet described in published literature, software must be made available to editors and reviewers. We strongly encourage code deposition in a community repository (e.g. GitHub). See the Nature Portfolio guidelines for submitting code & software for further information.

Data

Policy information about availability of data

All manuscripts must include a data availability statement. This statement should provide the following information, where applicable:

- Accession codes, unique identifiers, or web links for publicly available datasets
- A description of any restrictions on data availability
- For clinical datasets or third party data, please ensure that the statement adheres to our policy

ScRNA-seq data from this study have been deposited into the Gene Expression Omnibus (GEO) database under the accession number GSE193346 (https://www.ncbi.nlm.nih.gov/geo/query/acc.cgi?acc=GSE193346). The processed data has also been deposited to the UCSC cell browser and can be accessed via this link (https://cells-test.gi.ucsc.edu/?ds=mouse-dev-heart). The list of transcription factors was downloaded from the Mouse Genome Informatics database (http://

www.informatics.jax.org/mgihome/GO/project.shtml) with the Gene Ontology term 'DNA binding transcription factor activity'.

Human research participants

Policy information about studies involving human research participants and Sex and Gender in Research.

Reporting on sex and gender	*Use the terms sex (biological attribute) and gender (shaped by social and cultural circumstances) carefully in order to avoid confusing both terms. Indicate if findings apply to only one sex or gender; describe whether sex and gender were considered in study design whether sex and/or gender was determined based on self-reporting or assigned and methods used. Provide in the source data disaggregated sex and gender data where this information has been collected, and consent has been obtained for sharing of individual-level data; provide overall numbers in this Reporting Summary. Please state if this information has not been collected. Report sex- and gender-based analyses where performed, justify reasons for lack of sex- and gender-based analysis.*
Population characteristics	*Describe the covariate-relevant population characteristics of the human research participants (e.g. age, genotypic information, past and current diagnosis and treatment categories). If you filled out the behavioural & social sciences study design questions and have nothing to add here, write "See above."*
Recruitment	*Describe how participants were recruited. Outline any potential self-selection bias or other biases that may be present and how these are likely to impact results.*
Ethics oversight	*Identify the organization(s) that approved the study protocol.*

Note that full information on the approval of the study protocol must also be provided in the manuscript.

Field-specific reporting

Please select the one below that is the best fit for your research. If you are not sure, read the appropriate sections before making your selection.

☒ Life sciences ☐ Behavioural & social sciences ☐ Ecological, evolutionary & environmental sciences

For a reference copy of the document with all sections, see nature.com/documents/nr-reporting-summary-flat.pdf

Life sciences study design

All studies must disclose on these points even when the disclosure is negative.

Sample size	About 120,000 cells were profiled in 140 samples (covering most heart developmental stages), which means about 850 cells per samples. This targeted number of cells per sample is expected to cover all major cardiac cell types in each chamber.
Data exclusions	Low quality cells, and MULTI-seq barcode staining negative cells and multiplets.
Replication	The wildtype scRNA-seq datasets were generated in CD1 and C57BL/6 mouse strains. The two datasets were highly reproducible based on the UMAP plots. The mutant Wt1 and Tbx18 datasets were also profiled twice with the samples at certain stages were overlapped. The repeated datasets were highly consistent between replicates.
Randomization	For scRNA-seq experiments, the wild type embryos and neonatal mice were selected randomly. All Wt1 and Tbx18 mutant embryos were used after genotype, and their control embryos were selected randomly after genotype.
Blinding	Blinding is impossible or not relevant in this study. For the scRNA-seq of wild type mice, it's unnecessary to be blind. For the scRNA-seq of mutant mice, we need the genotype information before performing the experiments, and we used all mutant embryos from each litter in the experiments.

Reporting for specific materials, systems and methods

We require information from authors about some types of materials, experimental systems and methods used in many studies. Here, indicate whether each material, system or method listed is relevant to your study. If you are not sure if a list item applies to your research, read the appropriate section before selecting a response.

Materials & experimental systems

n/a	Involved in the study
☐	☒ Antibodies
☒	☐ Eukaryotic cell lines
☒	☐ Palaeontology and archaeology
☐	☒ Animals and other organisms
☒	☐ Clinical data
☒	☐ Dual use research of concern

Methods

n/a	Involved in the study
☒	☐ ChIP-seq
☒	☐ Flow cytometry
☒	☐ MRI-based neuroimaging

Antibodies

Antibodies used

Alex488-pHH3 (Abcam ab197502), cTNT (Abcam, ab45932), Vimentin (Novus Biologicals, NB300-223SS), CD31 (BD, 550274), and ALDH1A2 (Sigma, HPA010022), TGFB3 (R&D, MAB243SP), and cTNT (Thermo Fisher, MA512960).

Validation

Mouse monoclonal to Histone H3 (phospho S10) (Alexa Fluor® 488) [mAbcam 14955] (ab197502) was ordered from Abcam. The antibody was validated in Human and predicted to work in Mouse. The non-conjugated antibody [mAbcam 14955] was reported to work in Mouse, Human, Drosophila melanogaster, Recombinant fragment. 179 references on Abcam website have been found to successfully use the non-conjugated antibody.

cTNT (ab45932) was ordered from Abcam. The antibody was made in rabbit and predicted to work in multiple species but not including mice. However, based on our results, we saw specific signal in cardiomyocytes but not other cardiac cell types such as endothelial cells (Fig S21), indicating the antibody works in mice in immunofluorescence staining.

Vimentin antibody (NB300-223SS) was ordered from Novus Biologicals. It's a polyclonal antibody made in chicken and predicted to work in multiple species including mouse. There are 55 publications on this antibody listed on their website. Importantly, it has been reported to work in mouse in this literature (PMID:33675257).

CD31 antibody (BD, 550274) was ordered from BD. It was made in Rat using an antigen from 129/Sv mouse-derived endothelioma cell line tEnd.1. According to multiple literature including some studying heart development (PMID: 24278332), this antibody works in mouse.

ALDH1A2 (Sigma, HPA010022) was ordered from Sigma. It is a polyclonal antibody made in Rabbit by using antigen Retinal dehydrogenase 2 recombinant protein epitope signature tag (PrEST). It was reported to work in human. According to our staining results, we observed that the antibody specifically stained the heart outer layer cells (epicardium) in wild type hearts, indicating the antibody works in mice tissue as well.

TheTGFB3 antibody was ordered from R&D. The antibody can detect TGF-beta 3 from multiple species in direct ELISAs and Western blots. In Western blots, less than 25% cross-reactivity with recombinant human (rh) TGF- beta 1.2 and rhTGF-beta 2 is observed, and less than 2% cross-reactivity with recombinant amphibian TGF-beta 5 and recombinant human TGF-beta 1 is observed. Neutralizes the biological activity of TGF-beta 3 but not TGF-beta 1, TGF-beta 2, or TGF-beta 5.

Cardiac Troponin T Antibody (Thermo Fisher, MA512960) purchased from Invitrogen Catalog # MA5-12960,monoclonal mouse/IgG1. Species validated in dog, rat, hamster, zebrafish, mouse, human.45 Publications validated for Immunocytochemistry (ICC),7 Publications validated for Immunofluorescence (IF).Expression of Cardiac Troponin T was observed specifically in heart tissue and was negative for skeletal muscle and lung tissue in western blot.

Animals and other research organisms

Policy information about studies involving animals; ARRIVE guidelines recommended for reporting animal research, and Sex and Gender in Research

Laboratory animals

wildtype mice with CD1 and C57BL/6N strain; The Wt1 and Tbx18 mutant mice have mixed strains. The mouse embryos in the breedings ranging from E9.5 to P9 were profiled in the study.

Wild animals

Provide details on animals observed in or captured in the field; report species and age where possible. Describe how animals were caught and transported and what happened to captive animals after the study (if killed, explain why and describe method; if released, say where and when) OR state that the study did not involve wild animals.

Reporting on sex

As the gender in mouse embryos and neonatal mice are not obvious, we used them in the experiment without paying attention to their gender.

Field-collected samples

For laboratory work with field-collected samples, describe all relevant parameters such as housing, maintenance, temperature, photoperiod and end-of-experiment protocol OR state that the study did not involve samples collected from the field.

Ethics oversight

All animal experiments in the study were approved by the University of Pittsburgh Institutional Animal Care and Use Committee (IACUC).

Note that full information on the approval of the study protocol must also be provided in the manuscript.
